# Applications of a Novel Clustering Approach Using Non-Negative Matrix Factorization to Environmental Research in Public Health

**DOI:** 10.3390/ijerph13050509

**Published:** 2016-05-18

**Authors:** Paul Fogel, Yann Gaston-Mathé, Douglas Hawkins, Fajwel Fogel, George Luta, S. Stanley Young

**Affiliations:** 1 Independent Consultant, Paris 75006, France; paul_fogel@hotmail.com; 2 YGM Consult, CEO, Paris 75015, France; yann.gaston.mathe@gmail.com; 3 School of Statistics, University of Minnesota, Minneapolis, MN 55455, USA; dhawkins@umn.edu; 4 Institute Louis Bachelier, Paris 75002, France; fogel@di.ens.fr; 5 Department of Biostatistics, Bioinformatics, and Biomathematics, Georgetown University, Washington, DC 20057, USA; george.luta@georgetown.edu; 6 CGStat, CEO, Raleigh, NC 27607, USA

**Keywords:** SVD, PCA, NMF, K-means

## Abstract

Often data can be represented as a matrix, e.g., observations as rows and variables as columns, or as a doubly classified contingency table. Researchers may be interested in clustering the observations, the variables, or both. If the data is non-negative, then Non-negative Matrix Factorization (NMF) can be used to perform the clustering. By its nature, NMF-based clustering is focused on the large values. If the data is normalized by subtracting the row/column means, it becomes of mixed signs and the original NMF cannot be used. Our idea is to split and then concatenate the positive and negative parts of the matrix, after taking the absolute value of the negative elements. NMF applied to the concatenated data, which we call PosNegNMF, offers the advantages of the original NMF approach, while giving equal weight to large and small values. We use two public health datasets to illustrate the new method and compare it with alternative clustering methods, such as K-means and clustering methods based on the Singular Value Decomposition (SVD) or Principal Component Analysis (PCA). With the exception of situations where a reasonably accurate factorization can be achieved using the first SVD component, we recommend that the epidemiologists and environmental scientists use the new method to obtain clusters with improved quality and interpretability.

## 1. Introduction

Let us consider the number of emergency hospital admissions in several US communities for specific diseases, e.g., cardiovascular disease (CVD), myocardial infarction (MI), and congestive heart failure (CHF). A pattern of admission causes may be characterized by unusually high and/or low counts for some of the possible causes. A specific community may have a high similarity with a particular pattern, e.g., high CVD and low MI, a somewhat lower similarity with the opposite pattern, e.g., low CVD and high MI, and negligible similarities with other patterns. As such, an admission pattern can be thought of as the pattern of an *archetypal community*, in which all admission causes have average count levels, except for the ones that are unusually high /low and characterize the pattern itself. What if a community’s pattern is actually a *mixture* of patterns of archetypal communities, rather than being similar to one specific pattern? Matrix factorization methods can address both possibilities. The data used for the analysis of such problems can be represented as a two-way table, e.g., communities as rows and admission causes as columns. Any arbitrary ordering of communities or admission causes such as alphabetical order will result in a two-way table of numbers as if they were drawn at random. By contrast, separating the data set into blocks consisting of communities sharing a similar admission pattern will result in a two-way table that will facilitate the description of the data. The Non-negative Matrix Factorization (NMF) can be used to perform this operation. Enforcing the non-negativity constraint ensures that each community’s admission pattern can be modeled as an additive mixture of admission patterns of archetypal communities, where both the mixture coefficients and the archetypal patterns are simultaneously estimated. Comparing the mixture coefficients separates the two-way table into blocks of communities sharing a particular admission pattern. Within each block, communities can be sorted by their level of similarity with the admission pattern associated with the block. A similar scheme leads to the clustering and reordering of the various admission causes. The top ranked causes within each block constitute the main features of each pattern, making it easier to describe/summarize the blocks, with the resulting ordered matrix being conveniently represented by a colored heatmap [1].

By its nature, this clustering method is designed to focus on the large values within each block—where the corresponding communities have the largest mixture coefficients. One limitation of this strategy is that the communities with count levels close to average levels for all but a particular cause with a very low count, may be assigned at random to one or another pattern, when in fact it may be desirable to separate them from the rest of the communities into specific admission patterns—characterized by very low counts for certain admission causes. Our idea is to: (i) consider the normalized matrix of counts, where each entry can be either positive or negative, depending on whether the underlying count is unusually high or low; (ii) split the matrix into its positive and negative parts; (iii) concatenate both parts of the matrix after taking the absolute value of the negative elements; (iv) perform the NMF of the resulting matrix; (v) generate ordered clusters, which by construction correspond to either high or low counts from the original matrix. 

We call the new method PosNegNMF. Noteworthy, any mixed sign matrices can be analyzed by using PosNegNMF, despite not being non-negative. There are connections between clustering methods and matrix factorization methods. For example, the result of a K-means clustering run can also be written as a matrix factorization, where the mixture coefficients become cluster membership indicators and the archetypal patterns are given by the cluster centroids. The semi-NMF approach, where archetypal patterns typically have entries with both positive and negative signs, and only the mixture coefficients are nonnegative, is a relaxation of K-means clustering. This variant of NMF has been shown to be more accurate than K-means on a number of datasets in retrieving known clusters [2]. Since PosNegNMF can also be mathematically formulated as a semi-NMF of the two-way table, as will be shown later in the paper, it inherits its clustering performance. Nevertheless, both K-means and semi-NMF allow for clustering of *either* observations *or* variables, however in a non-simultaneous way, whereas our approach allows for a *simultaneous* clustering of observations *and* variables, yielding comprehensive heatmaps.

We illustrate this novel clustering approach using two datasets from the research area of public health. The approach is compared with the standard NMF approach, K-means and clustering based on Singular Value Decomposition (SVD).

## 2. Materials and Methods

### 2.1. Data Sets

Two data sets were analyzed:

(i) The number of emergency hospital admissions for cardiovascular disease (CVD), myocardial infarction (MI), congestive heart failure (CHF), respiratory disease, and diabetes were collected in 26 US communities, for the years 2000–2003 [3].
(ii) The Compressed Mortality File (CMF)—a county-level national mortality and population database spanning the years 1968–2010. The table contains death counts for 13 age categories.

### 2.2. Methods

#### 2.2.1. Non-Negative Factorization of a General Matrix

Let us consider a matrix **V** that contains only non-negative entries. **V** can be approximated by a sum of *k* rank-1 bilinear forms **V** = **∑** **w*_q_***·**h*_q_***^T^, more conveniently written as **V** = **WH**^T^, with **W** = [**w*_q_***] _1 ≤ *q* ≤ *k*_ and **H** = [**h*_q_***] _1 ≤ *q* ≤ *k*_. The vectors **w*_q_*** and **h*_q_*** are referred to as the *components* of the factorization model. The NMF of **V** can be used to obtain **W** and **H**, and thereby guarantees that all elements of **W** and **H** are non-negative—by contrast with the ordinary SVD of **V**, which returns **W** and **H** with mixed signs. Consider now a general matrix **V**, which we would like to analyze with a NMF approach. If **V** is of mixed signs, a preliminary transformation is required to satisfy the non-negativity requirement. Two approaches can be applied. The first approach is a novel approach that is referred to as the PosNegNMF approach:

(i) Split **V** into the positive and negative parts : **V** = **V**_+_ − **V**_−_ where **V**_+_ contains the positive entries, other entries being replaced by 0, and **V**_−_ contains the absolute values of the negative entries, other entries being replaced by 0.
(ii) When the rows are the observations and the columns are the variables, use the horizontally concatenated matrix **V_PN_** in order to give equal weight to low and high features while characterizing the clusters of observations.
(iii) Apply the NMF clustering on the concatenated matrix: **V_PN_** = **WH**^T^ (note that writing **V** = **W(H**_+_ − **H**_−_**)**^T^ where **H** = [**H**_+_ **H**_−_] corresponds to the semi-NMF model).

The second approach is referred to as the affine NMF approach:

(i) Substract the minimum of each column: **V** = **V_0_** + **baseline** [1].
(ii) Apply the NMF clustering to **V_0_**.

A variety of algorithms can be used to obtain the NMF model components **W** and **H**. The simplest method uses multiplying updating rules [4] to minimize the sum of squares of the elements of **V** − **WH^T^**, which we will refer to as the residual sum of squares (Appendix A). Note that in contrast to the analysis of **V**_PN_, which includes all information contained in **V**, the analysis of **V_0_** disregards the baseline, and so it includes only a part of the information contained in **V**. We will show in the results section that this loss of information may have dramatic consequences on the quality and interpretability of the clustering results.

#### 2.2.2. NMF Clustering and Reordering

Data clustering methods such as K-means can be applied within the space generated by the column vectors of **W** to cluster observations, as traditionally done with SVD-based methods, such as the latent semantic indexing method used in document clustering [5]. The simple clustering scheme described in the introduction is based on the direct link of each homogeneous block in **V** with a particular column in **W**, which has the largest elements point to this block. However, neither approach is independent of the chosen scaling system, which is arbitrary. To address this problem, component leverages can be calculated. The component leverage represents the ability of a row or column to exert specifically an influence on a particular component—without a corresponding increase in the influence on other components. Leverage column vectors have all elements in the interval (0, 1). They are strongly correlated with the NMF column vectors **w_q_** and **h_q_**, however much less affected by the choice of a scaling system. Technical details for the calculation of leverages are given in Appendix B.

#### 2.2.3. Stability and Specific Clustering Contribution of NMF Clusters

The clustering method that we consider in this paper operates in a rather dichotomic way as samples must be assigned only to one cluster, even when the assignment may be ambiguous. The stability of NMF clusters is evaluated by applying a resampling scheme. The validation of the identified clustering structure is the most difficult and challenging part of cluster analysis, and it goes far beyond the scope of this paper. We propose a simple, internal validation tool—which does not rely on external information such as known group membership: the specific clustering contribution (SCC) criterion is derived from the concept of entropy [6] to provide a quantitative assessment of the within-cluster homogeneity and the between-clusters heterogeneity. Details for the calculation of the stability and SCC are given in Appendix C. For both indicators, the result is a number that is in the interval (0, 1).

#### 2.2.4. Rank of the NMF Factorization

Depending on the chosen factorization rank, the clusters may appear more or less stable, and consequently providing additional criteria for its determination. The factorization rank *k* can be selected through the analysis of the sum of squares of the residual matrix **V** − **WH^T^**, where *k* takes values from 1 up to a large value (e.g., half the number of rows or columns), in combination with the analysis of cluster stability. The plot of each criterion as a function of *k* is called a scree plot. An optimal *k* should correspond to both a low residual sum of squares and a high cluster stability. Additionally, the scree plot of the cluster SCC can be used to confirm the choice based on the first two criteria. Ultimately, selecting *k* is a difficult decision to make, and it is an important step on the path to a better understanding of the nature of **V**.

#### 2.2.5. Normalization of Contingency Tables

The cells of a contingency table contain counts, which are all non-negative numbers, suggesting that NMF could be applied directly to the table. However, very different totals for rows or columns—as it often occurs in such tables—may result in highly heterogeneous variances, in sharp contrast to the error model behind NMF which assumes a homogeneous distribution across cells—at least when the algorithm attempts to minimize the residual sum of squares, similar to SVD. This problem is considered by Correspondence Analysis (CA) which uses the normalized matrix of contingency ratios. We briefly recall the main steps involved in this approach:

(i) For each cell, the contingency ratio is calculated by forming the ratio of the true count over the expected count—assuming the independence of rows and columns
(ii) Further normalization steps include the subtraction of the expected ratio under the assumption of independence (=1), yielding a mixed signs matrix, and a subsequent scaling of rows and columns to ensure homogeneous cell variances.
(iii) The SVD is applied to the normalized matrix.
(iv) A biplot based on the first two SVD components is then performed, allowing for a simultaneous clustering of the rows and columns of the table, which we will refer to as the SVD clustering. Note that PCA clustering refers to the same approach, since PCA’s eigen vectors are the column singular vectors [7].

More details can be found in [8]. The normalized matrix can alternatively be analyzed using the PosNegNMF clustering.

### 2.3. Software

We used a software in the form of an addin JMP (SAS Institute, Cary, NC, USA). The addin was developed in the JSL language and can be downloaded from Supplementary 1.

## 3. Results

### 3.1. Hospital Admissions Data

The objective of the analysis is the identification of archetypal patterns and the subsequent clustering of cities, seeking clusters characterized by relatively low or high counts for one or more causes of hospital admission.

#### 3.1.1. PosNegNMF Clustering

**Figure 1:**
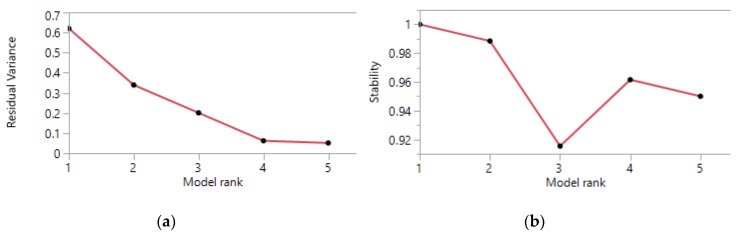
(**a**) Residual sum of squares; (**b**) Row clustering stability.

The PosNeg splitting scheme was applied to the normalized matrix of contingency ratios. The evaluation of the scree plot for the residual sum of squares (Figure 1a) and the clustering stability (Figure 1b) indicated that four component vectors provide a small residual variance (it almost stops decreasing when considering an additional component) and a good stability (it increases back to a level >0.95 from a level <0.92 with a three components model). The criteria were calculated for up to five components—the number of admission categories before the matrix was split.

**Figure 2:**
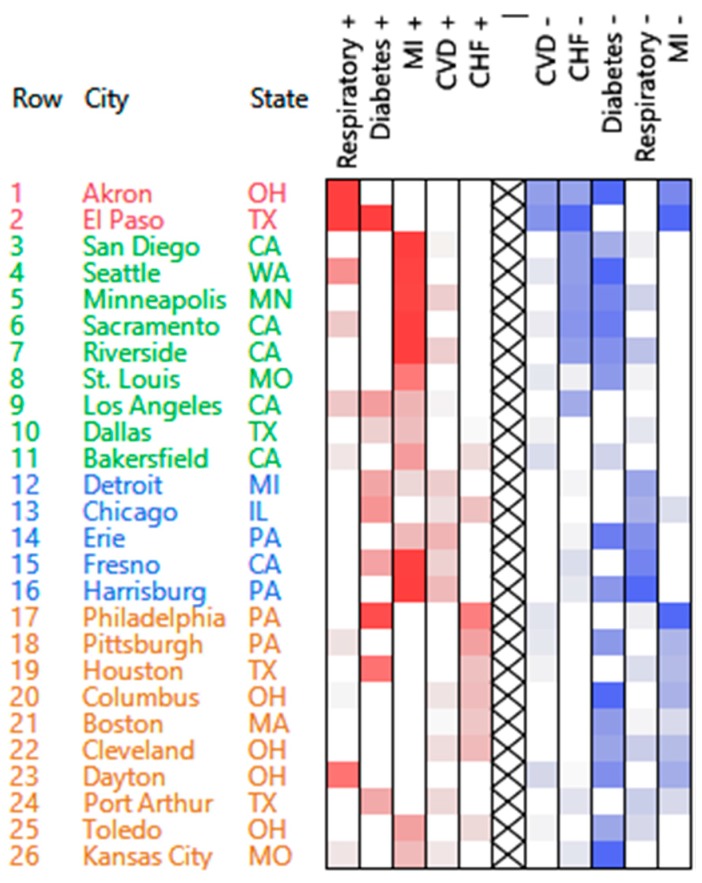
NMF clustering and re-ordering of hospital admissions by city and cause. Red: High count; Blue: Low count.

The count patterns are presented on the heatmap of the reordered rows and columns of the normalized contingency table (Figure 2). These patterns are characterized by high and/or low counts levels for each cause of hospital admission. Specifically, each row of the heatmap provides the counts of hospital admissions of the corresponding city using the color coding of the cells (*i.e.*, red = high, blue = low). High/low counts of hospital admissions appear on the left/right side of the heatmap, respectively—the suffixes “+” and “−“ were added to variable names to represent the positive and negative parts. The four clusters derived from the four model components are identified through the colors given to the city labels. Noteworthy, the third cluster (Detroit, Chicago, *etc.*) is mostly characterized by a lower number of hospital admissions due to respiratory disease, illustrating the role of low values for PosNegNMF clustering. 

The summary characterization of each cluster is given in Table 1. All variables were simultaneously clustered. Note that the positive and negative parts of a given variable are included in different clusters, e.g., the positive part of the respiratory cause is included in the first cluster, while the negative part is included in the third cluster.

Noteworthily, a K-means clustering of the normalized matrix of contingency ratios yields 6 clusters—following the Cubic Clustering Criterion (CCC) on the choice of *k* which is used classically with K-means [9]. Three K-means clusters are very close to PosNeg clusters 2, 3 and 4. However, the other three clusters have only one member. Moreover, cities cannot be ordered within each cluster as we do with PosNeg NMF—since K-means returns only cluster membership indicators—and causes cannot be easily assigned to each cluster since they are not simultaneously clustered. This illustrates the advantages of using PosNegNMF over K-means, as it helps summarizing with a heatmap the hospital admission patterns across US cities. It would be interesting to investigate these results more thoroughly, for example by using city characteristics to predict cluster membership, but this would take us beyond the scope of this article.

#### 3.1.2. Affine NMF Clustering

**Figure 3:**
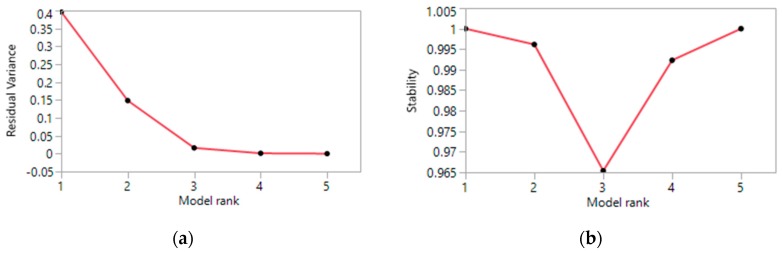
(**a**) Residual sum of squares; (**b**) Clustering stability.

For each cause of hospital admission, the minimum count over all cities for this particular cause was first subtracted out of the count of each city. The evaluation of the scree plots for the residual sum of squares (Figure 3a) and the clustering stability (Figure 3b) indicated—as for PosNeg NMF—that four component vectors provide both a small residual variance and a good stability.

**Figure 4:**
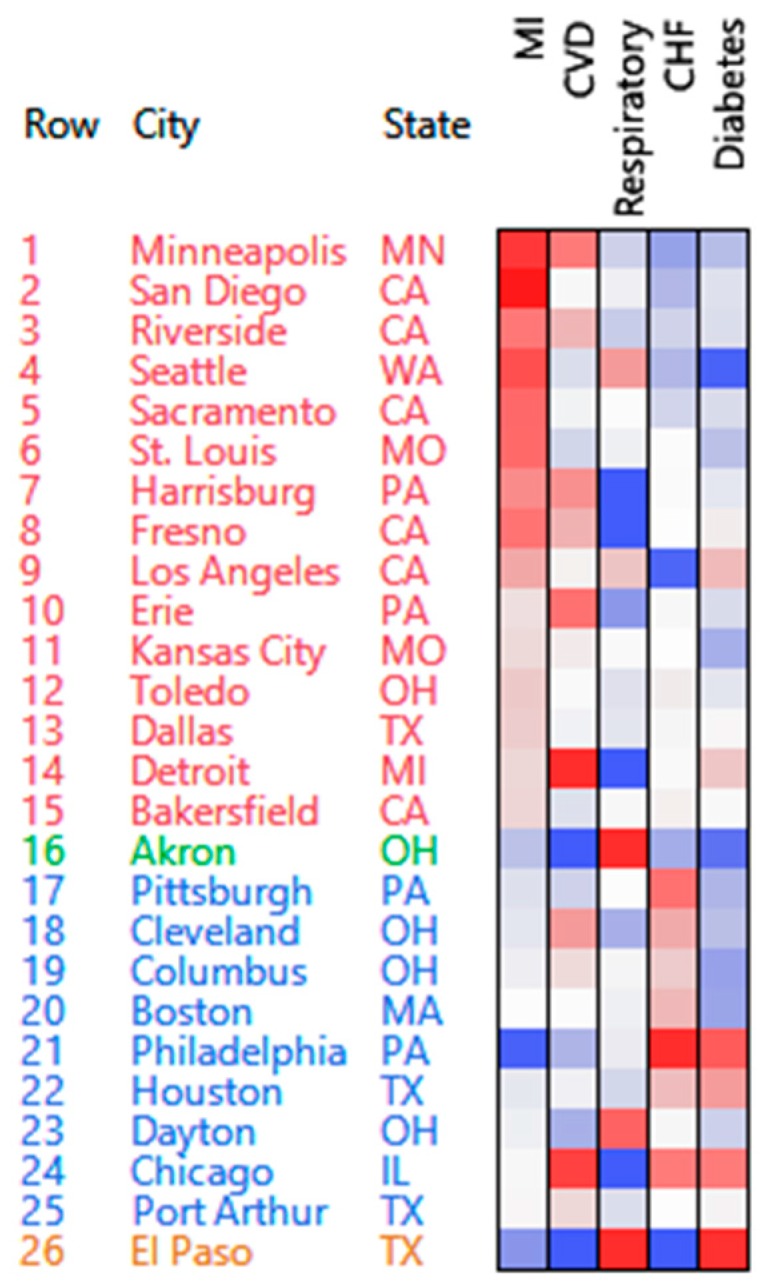
Affine NMF clustering. Red: High count; Blue: Low count.

The ordered heatmap and clusters obtained with the affine NMF approach are shown in Figure 4. The clusters produced by the affine NMF method are neither as clearly separated nor easier to describe/summarize as those produced by the PosNegNMF method. Specifically, the second and fourth cluster from the affine NMF method contain only one city each. As a consequence, extracting from this figure a summary characterization of the clusters as we did with PosNeg NMF in Table 1 appears difficult, apart from the very main features: high myocardial infarction (MI) in cluster 1, and high congestive heart failure (CHF) in cluster 3.

**Figure 5:**
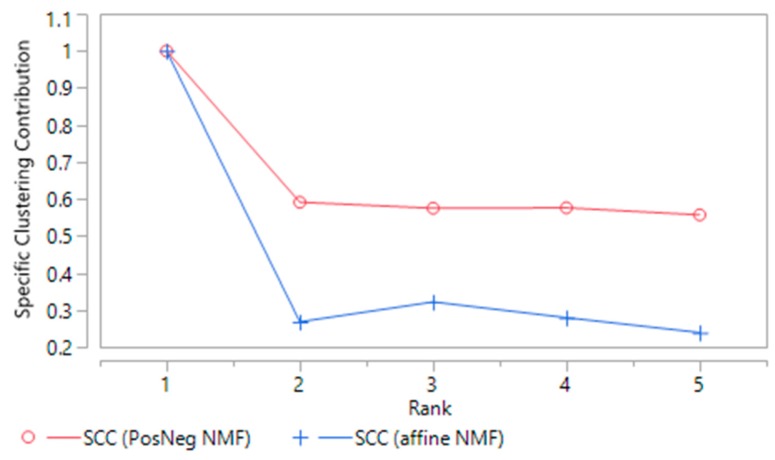
Specific clustering contribution of NMF clusters, PosNegNMF and affine NMF approaches.

The difference in clustering quality is further evidenced by the scree plots for the specific clustering contributions of the two clustering methods (Figure 5), where the SCC is uniformly higher when the PosNegNMF approach is used.

#### 3.1.3. Correspondence Analysis

**Figure 6:**
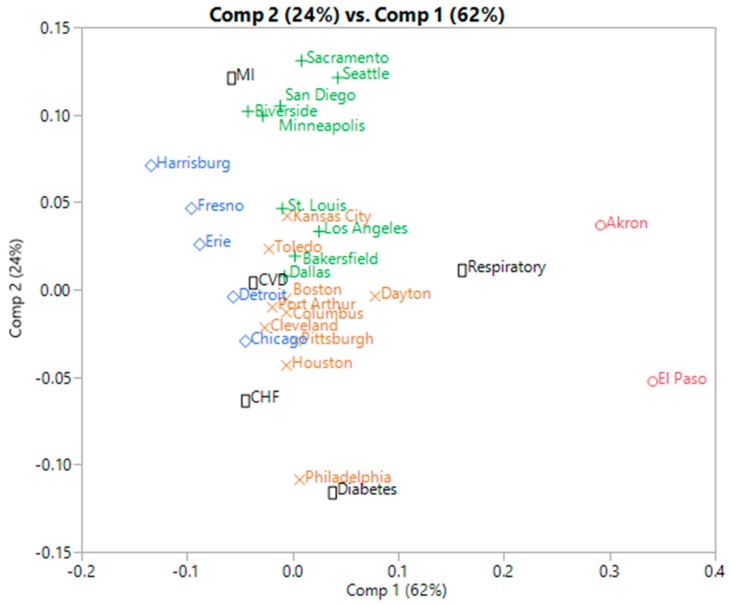
Correspondence analysis biplot of hospital admissions by city and cause (PosNegNMF clusters are represented by the city label colors).

The first two SVD components of the normalized contingency matrix allow for a simultaneous representation of cities and hospital admission causes. This kind of representation is called a biplot and is widely used when performing Correspondence Analysis (CA, Figure 6). We assess whether the clusters of cities and the admission patterns can be retrieved through this biplot. PosNegNMF clusters are presented using colored city labels, with the same colors as in the heatmap from 3.1.1. The proximities between clusters and causes of hospital admissions help the interpretation of the two axis of the biplot, e.g., Sacramento, Seattle and nearby cities, all have high levels of MI. However the corresponding detailed counts are not visible and they can only be inferred from these proximities, a process which is not reliable. For example, St Louis, Los Angeles, Bakersfield and Dallas (PosNegNMF cluster 2, green crosses) appear clustered on the biplot with Kansas City, Toledo, *etc.* (PosNegNMF cluster 4, brown × symbols) despite their relatively high level of MI, which is characteristic of cluster 2. This suggests that the biplot may be too restrictive, possibly because of being limited to the first two SVD components.

#### 3.1.4. Additional Remarks

The three methods (PosNeg NMF, Affine NMF, and Correspondence Analysis) deal with the communities of Akron, OH, and El Paso, TX, USA, in different ways. These communities are similar with respect to four out of the five admission causes, but they are different with respect to diabetes (El Paso is high with respect to diabetes, while Akron is low). The PosNeg NMF method reveals the similarities between these two communities while at the same time pointing to the unique difference between them. The Affine NMF method considers the two communities as two separate clusters due to their differences with respect to diabetes (and thereby completely ignores the similarity with respect to the other causes). Correspondence analysis plots these two communities on the right side of the biplot, somewhat distant from each other on the vertical axis. Since diabetes is plotted at the bottom of the biplot, it may be concluded that the distance between these two communities is due to diabetes (although this may require extensive expertise with correspondence analysis).

### 3.2. Compressed Mortality File

**Figure 7:**
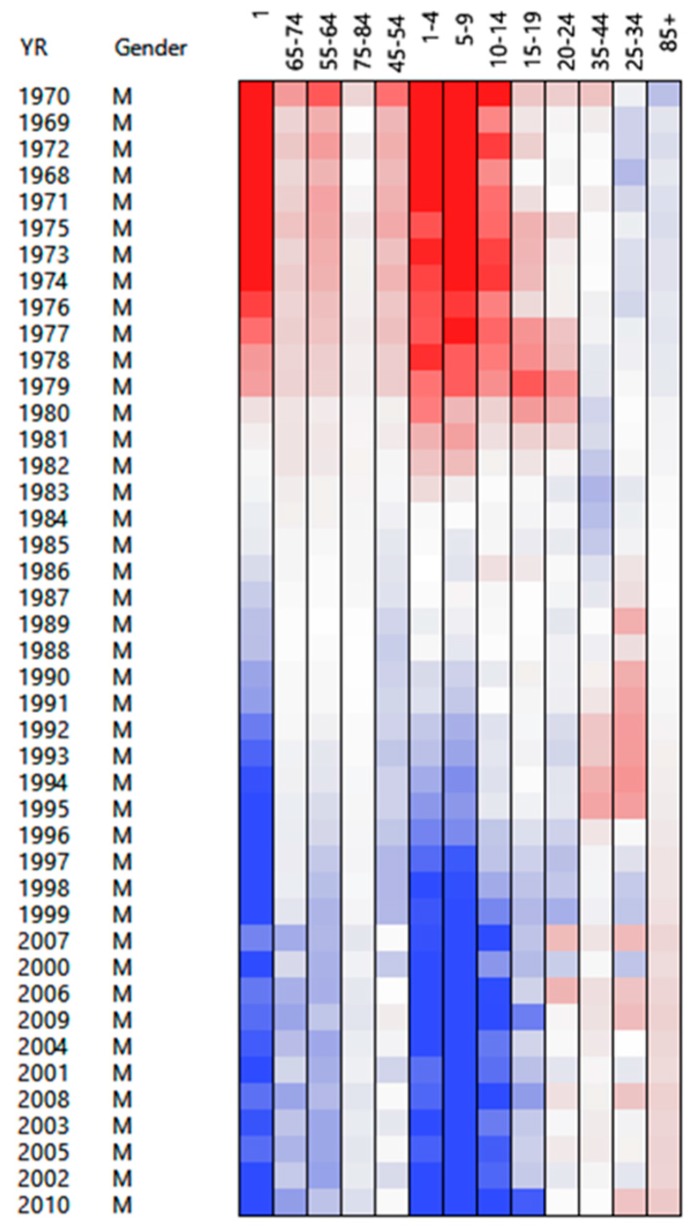
SVD reordering of the rows and columns of a life table. Red: High count; Blue: Low count.

We have presented an example where the PosNegNMF method outperforms the original NMF or CA/SVD methods. Nevertheless, whenever the studied matrix can be approximated well by a rank-1 bilinear form, using this new approach is not recommended. The Compressed Mortality File (CMF)—a county-level national mortality and population database spanning the years 1968–2010—will help illustrate this important point. The table contains death counts for males for 13 age categories. The analysis of the normalized matrix of contingency ratios shows that the second SVD component contributes only 6% of the overall variance. Thus, rows and columns can be reordered through the rank-1 bilinear approximation given by the first SVD component, revealing well-known trends in the pivot period marking the end of 20th century (Figure 7), such as more deaths being observed in the age category 85+, and less deaths are observed in younger age categories. Also as we enter the 21st century people tend to live longer. A sudden increase in deaths is observed in the younger age categories (25–44 years old) between 1985 and 1995 (AIDS outbreak), which tends to get back to normal after 1996 once the AIDS drugs come into wide use.

## 4. Discussion

### 4.1. Performance

Our experience suggests that the computational performance of the NMF algorithm is improved when the PosNeg transformed matrix is used, despite the fact that there are twice as many columns as in the original matrix. This is due to the smaller number of iterations for the algorithm to converge—suggesting a more stable solution. It has been shown that a version of the algorithm, using an Expectation-Maximization (EM) scheme [10], can yield a better approximation for sparse matrices. Such scheme seems appropriate for PosNegNMF, given the sparse nature of the transformed matrix.

### 4.2. Alternative Approaches

While NMF allows to simultaneously cluster rows and columns of the input matrix **V**, we could also use standard clustering methods to first cluster rows, and then columns of **V**. A rank-1 bilinear form approximation provides a natural way of performing these operations. Approximating the matrix **V** by **V** = **wh**^T^, where **w** and **h** are the row and column marker vectors respectively, then reordering the rows of **V** by the decreasing values of the elements of **w** and the columns by the decreasing values of the elements of **h** leads to a matrix with elements that tend to go from the largest in the top left to the smallest in the bottom right. The bilinear approximation also provides a way to cluster the matrix into internally homogeneous rectangles. Sorting the elements of **w** into descending order and separating them into *k* maximally homogeneous clusters leads to the reordered **V** having rows that are maximally homogeneous to the extent the bilinear approximation matches them well—the elements of the error matrix **V** − **wh**^T^ are small enough. Applying the same operation to separate the elements of **h** into *m* maximally homogeneous clusters does the same for the columns, while separating the elements of both the row and column markers will cluster the matrix **V** into *km* internally homogeneous blocks. This approach was used in Liu *et al*. [11] where a robust incomplete-data implementation of SVD has been used to obtain the approximants **w** and **h**. The segmentation of the row and column markers was carried out using the dynamic programming optimization method from Hawkins [12]. 

It may not always be sensible to permute the rows or columns of the matrix, for instance when one considers time series or geographic data, which are naturally ordered. In this setting a separation into rectangular blocks can still be performed by partitioning the array into homogenous. This approach, described in Hawkins [13], may not lead to explicit optimization algorithms but can be implemented using heuristic approaches. It can also be applied to more general matrix clustering problems during the second stage that follows the initial reordering of rows and columns using a bilinear approximation or other methods of reordering the rows and columns, such as spectral clustering [14,15,16]. Algorithms that try to retrieve such an order are generally called seriation algorithms (see [17] for a historical review).

### 4.3. Limitations

Our research is focused on improving the quality and interpretability of clusters. When the primary objective is to determine the compositions (the columns of **H**) of a mixture of true real-world sources, and their contributions (the columns of **W**) to observed samples (the rows of **V**), finding “the right solution”, and determining the uniqueness of the solution, are critical goals. These two problems are essentially different, along the lines of Shmueli [18], to explain—through cluster analysis—or to predict—estimates of mixture contributions. Whether PosNegNMF improves on existing mixture deconvolution methods will require further investigation. Positive Matrix Factorization (PMF) [19] has been mostly applied to mixture problems, such as determining the sources of atmospheric pollution. Both NMF and PMF approaches actually solve the same mathematical problem with very close algorithms. However, PMF is more oriented towards mixture deconvolution problems—yielding accurate mixture contributions, where as NMF is more oriented towards pattern discovery—yielding interpretable clusters. It should be noted that the PosNeg transformation generates a sparse matrix with at least half of the cells having zero counts. Obviously, matrices which are naturally sparse are not amenable to the PosNeg transformation.

## 5. Conclusions

We showed that the heatmap of the reordered rows and columns of a matrix, when properly normalized, can add insight to the SVD clustering produced by CA, in particular with respect to the interpretation of the biplot axes. We also showed that PosNegNMF clustering returns more homogenous clusters, in contrast to affine NMF clustering.

In the more general context of NMF, we have provided methodological innovations to address some of the known caveats of the NMF approach such as component scaling indetermination and the choice of an optimal NMF rank. The calculation of leverages allows for a component scale-free clustering of the rows or columns of a non-negative matrix, while the evaluation of the stability and specific clustering contribution of NMF clusters provide additional criteria for choosing an optimal NMF rank.

We have proposed and illustrated with two examples a novel clustering approach using NMF. With the exception of situations where a reasonably accurate factorization can be achieved using the first SVD component, the new method may be used by epidemiologists and environmental scientists to obtain clusters with improved quality and interpretability, compared to those obtained from commonly used clustering methods, such as K-means and the clustering produced by CA.

## Figures and Tables

**Table 1:** High and low counts by cluster.

Cluster	High Counts	Low Counts
1	Respiratory	CVD, CHF, MI
2	MI	CHF, diabetes
3	MI, CVD	Respiratory
4	CHF	Diabetes, MI

Cardiovascular disease (CVD), myocardial infarction (MI), congestive heart failure (CHF).

**Figure B1:**
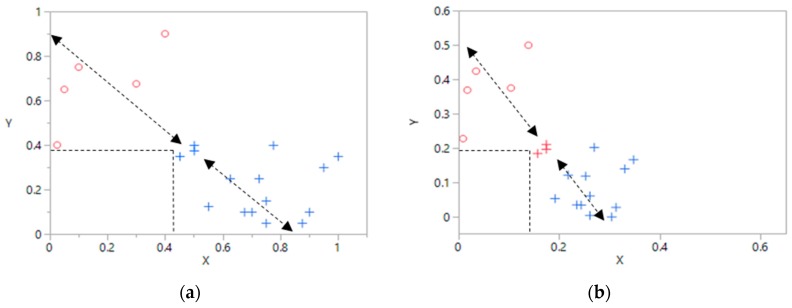
(**a**) Clustering in the original coordinate system; (**b**) Clustering using scaled coordinates.

**Figure B2:**
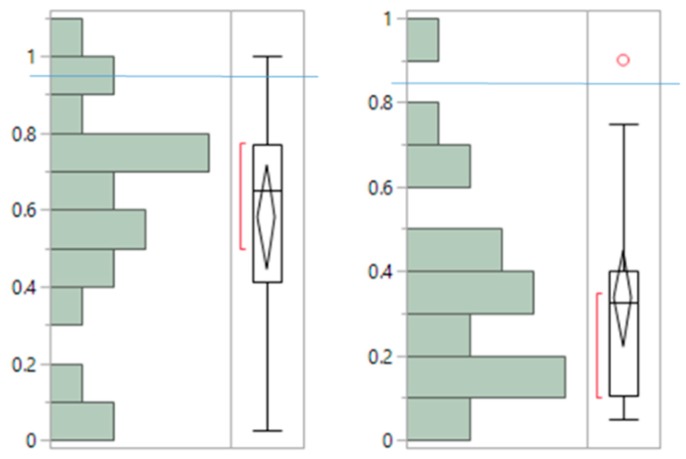
Estimation of a robust max on each component vector (blue line on top of each histogram).

## Supplementary Materials

The datasets used in this paper are available online at http://www.mdpi.com/1660-4601/13/5/509/s1.

## Abbreviations

The following abbreviations are used in this manuscript:

CA	Correspondence Analysis
NMF	Non-Negative Matrix Factorization
PMF	Positive Matrix Factorization
SVD	Singular Value Decomposition
CVD	cardiovascular disease
MI	myocardial infarction
CHF	congestive heart failure
SCC	Specific clustering contribution

## Appendix A: Estimation of the NMF Model Components W and H

For a given rank *k*, we start by initializing the elements of the nonnegative matrices **W** and **H**:

**W***iq* > 0, **H***qj* > 0, ∀ *i*, *j*, *q*, where *i*, *j*, and *q* are the row, column, and component indexes.

Then we apply the following multiplicative update rules until the difference between two iterations is small:

**W***iq* ← **W***iq* (**VH**)*iq*/(**WH**^T^**H**) *iq*, ∀ *i*, *q*

**H***jq* ← **H***jq* (**W**^T^**V**)*qj*/(**W**^T^**WH**^T^) *qj*, ∀ *j*, *q*.

At each iteration, we update the current elements of **W** and **H** using specific multiplicative factors that relate to the current quality of the intended approximation, see Lin, 2005 [20] for further details regarding the properties of this algorithm and extensions using projected gradient methods. An affine NMF variant of these updating rules can be found in Laurberg, 2007 [21], however this variant applies only to non-negative matrices, whereas the affine approach that uses the minimum of each column applies to mixed signs matrices. Note that the NMF model components are not ordered: the order in which they appear in the factorization can change, depending on the initialization and algorithm used.

## Appendix B: Calculation of Leverages

The NMF squared elements of the columns of **W** or **H** are typically constrained to sum to unity as a convenient way of eliminating the degeneracy associated with the invariance of **WH** under the transformation **W** → **WΛ**, **H** → **Λ**^−1^**H**, where **Λ** is a diagonal matrix defining a particular scaling system. It should be noted that the sum to unity constraint is arbitrary. We will show that the choice of a particular system can affect the clustering process.

Assume that samples are mixtures of two archetypal samples **H**_1_ and **H**_2_. In Figure B1a, the sample coordinates (*x*, *y*) correspond to the mixture coefficients in a two-component model: Sample = *x***H**_1_ + *y***H**_2_. The red circles correspond to samples with *y* > *x*, thus more similar to **H**_2_; the blue crosses correspond to samples with *y* < *x*, thus more similar to **H**_1_. Now, the *x* and *y* vectors were scaled by the square root of their respective sum of squares, yielding a new coordinates system. When this system is used, three samples originally closer to **H**_1_ appear wrongly clustered with samples originally closer to **H**_2_ (Figure B1b, red crosses). Indeed, for these particular samples, in this coordinates system, *y* > *x*, where as in the original system, *y* < *x*. If the distance to **H**_1_ or **H**_2_ is now defined by the Euclidean distance to the extremity of each axis (represented by an arrow), the clustering remains unchanged in either coordinates system.

This distance, which appears to be independent of the chosen scaling system, can be easily extended to more than two component vectors. For each observation *i*, the distance to each profile vector **H***q* is defined by:

(B1) $$distance\left(i,Hq\right)={\left(\underset{j}{\max}\left(W\left(j,q\right)\right)-W\left(i,q\right)\right)}^{2}+\overset{k}{\underset{r\neq q}{\sum }}W{\left(i,r\right)}^{2}$$

The *leverage* can be directly derived from the distance:

(B2) $$leverage\left(i,Hq\right)=\text{exp}\left(-\frac{distance\left(i,Hq\right)}{2\;\cdot \;\underset{j}{\text{mean}}\left(distance\left(j,Hq\right)\right)}\right)$$

The leverage of each variable can be defined in a similar way. By construction, *leverages* are well correlated with the column vectors of **W** or **H**, and are in the interval (0, 1). They are little affected by the chosen scaling system, thus allowing for a more reliable clustering.

Now, the distance to each column vector **H***q* is a function of $\underset{j}{\max}\left(W\left(j,q\right)\right)$, thus it can be severely affected by outliers. The following iterative algorithm allows for estimating a robust maximum:

1. Initialize the robust estimate by the maximum of each component.
2. For each vector component *q*:
  - For each row *i* of **W**, calculate the probability *p* (*i*, *q*) and the *row score* (Equations (C2) and (C1) respectively, Appendix C).
  - Force the row score to 0 if *p* (*i*, *q*) < 1/*k*.
  - Update Robust Max(*q*) by the weighted mean of **W**(*i*, *q*), where the mean is taken over all samples satisfying $W\left(i,q\right)>{\underset{j}{95}}^{th}quantile\left(W\left(j,q\right)\right)$, and the weights are the row scores. The idea is that rows with higher row scores should weigh more on the max estimation.
  - Replace all **W**(*i*, *q*) > Robust Max(*q*) by Robust Max(*q*).
3. Repeat 2. until convergence.

Going back to the simulation example of Appendix A, the maximum appears above the horizontal blue line representing the robust maximum in the histograms of each column vector (Figure B2). Note that on the second component, the maximum was also identified as an outlier by a standard criterion based on the interquartile range.

## Appendix C: Stability and Specific Clustering Contribution of NMF Clusters

Stability: The stochastic nature of most clustering algorithms has provided methods for evaluating the consistency and robustness of their performance [22]. The central idea is to perform numerous runs of the algorithm, starting from different random initializations. On each run, the algorithm groups the rows into clusters, allowing for the calculation of the frequency at which two different rows fall into the same cluster. In contrast to random initialization, we first calculate a pseudo-unique NMF solution based on SVD initialization, which is itself unique [23]. The rows of **V** are resampled with replacement and the rows of **W** are resampled in exactly the same way as in **V**. **H** is then re-estimated, while the resampled **W** remains fixed. Column leverages are re-estimated after each run of this resampling scheme, giving rise to different clusters of columns, and the frequency at which a column falls into a particular cluster can be calculated. The higher the frequency, the more stable will be the column with respect to this cluster. Conversely, the updated **H** can be used to re-estimate **W**, which gives rise to different clusters of rows. Similarly, the frequency at which a row falls into a particular cluster can be calculated.

Specific Clustering Contribution: For each row of **W**, we define the row score for the *i*th row as:

(C1) $$Row\;score\left(i\right)=1+\frac{1}{\log_{2}\left(k\right)}\overset{k}{\underset{q=1}{\sum }}p\left(i,q\right)\log_{2}\left(p\left(i,q\right)\right)$$

where *p*(*i*, *q*) is the probability that the *i*-th row contributes to cluster *q*, *i.e.*,:

(C2) $$p\left(i,q\right)=W\left(i,q\right)/\overset{k}{\underset{r=1}{\sum }}W\left(i,r\right)$$

The *row score* is a real value within the interval (0, 1). The higher the row score value, the more component-specific the corresponding rows [6]. The mean row score over all rows of **W** provides an overall indicator of the Specific Clustering Contribution (SCC).
