# Supplementary material for: Applications of a Novel Clustering Approach Using Non-Negative Matrix Factorization to Environmental Research in Public Health
Source: Int J Environ Res Public Health. 2016 May 18;13(5):509. doi: 10.3390/ijerph13050509 (PMC4881134; doi:10.3390/ijerph13050509)
Supplement: Supplementary file 1 [file ijerph-13-00509-s001.zip › irMF700.zip!/irMF700.pdf]

Readme irMF V7.0.0

Revision date: May 11th, 2016

Contact Information: genetree@bellsouth.net, paul_fogel@hotmail.com

Contents

TABLE OF CONTENTS

1 Installation and Update 3

1.1 Installation 3

1.2 Update 4

1.3 Uninstallation 4

1.4 Professional versus Restricted mode 5

2 Data file format 5

3 Subset selection 6

4 irMF main dialog tab 9

5 Other dialog tabs 15

5.1 irMF+ 15

5.1.1 Robust NMF 16

5.1.2 NMF variants (irMF PRO) 18

5.2 Cell plot 19

5.3 Advanced 21

5.4 Utilities 23

5.5 Build 26

5.6 Tools 28

5.7 Preferences 29

5.8 NAVIGATION 31

6 Outputs 31

7 Notes 32

8 A small tutorial 32

References 41

1 Installation and Update

1.1 Installation

Note: irMF V6.0.1 operates as a SAS JMP script (version 9.0.0 or higher).

irMF 6.0.1 is encapsulated in a jmp addin setup file named `irMF.jmpaddin`. Simply double-click this file

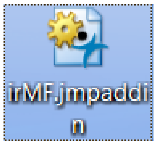

and the add-in will automatically install. A dialog will pop up to confirm installation.

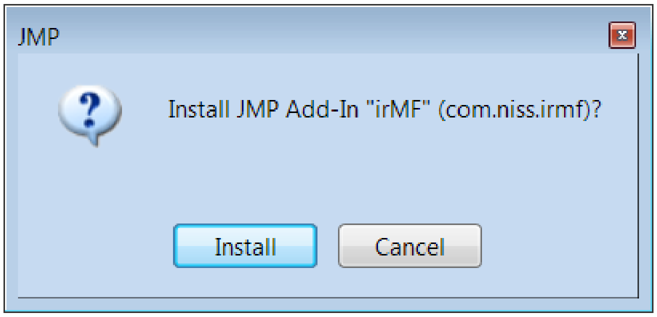

You should now see the irMF icon at the end of the `Analyze` tool bar.

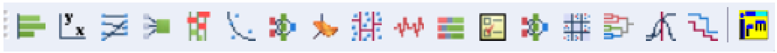

irMF option also appears in jmp main menu at the end of the `Analyze` menu.

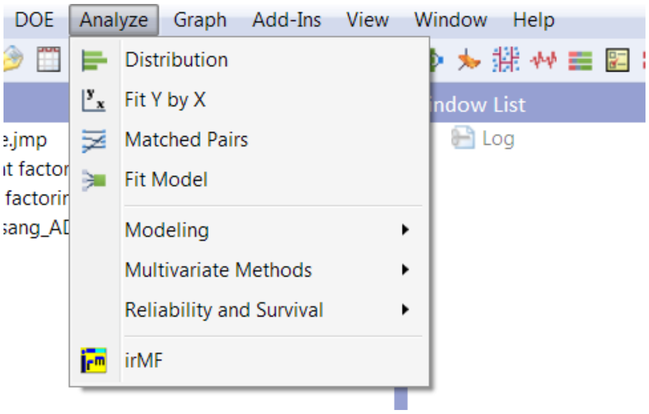

1.2 Update

To install an update of irMF, follow the same procedure as for installation. A dialog will pop-up to confirm reinstallation.

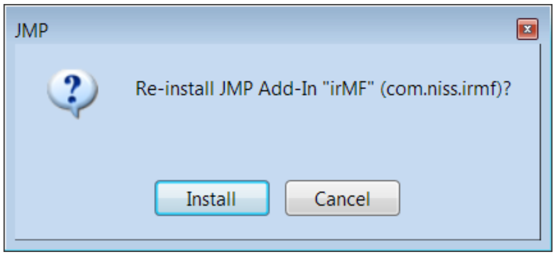

1.3 Uninstallation

If you wish to uninstall irMF, select the menu *Add-ins* (under the *View* option in JMP main

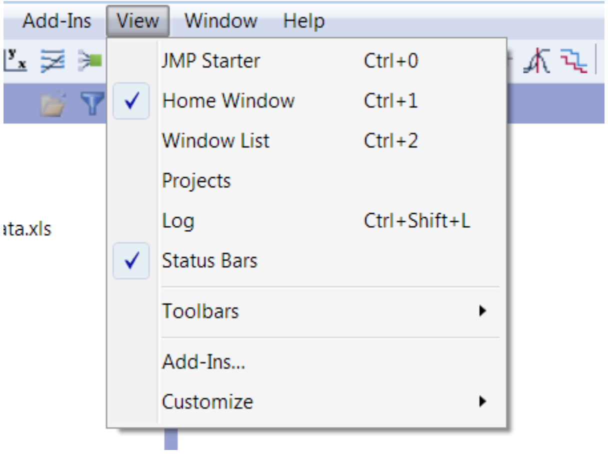

menu).

A list of registered add-ins is displayed:

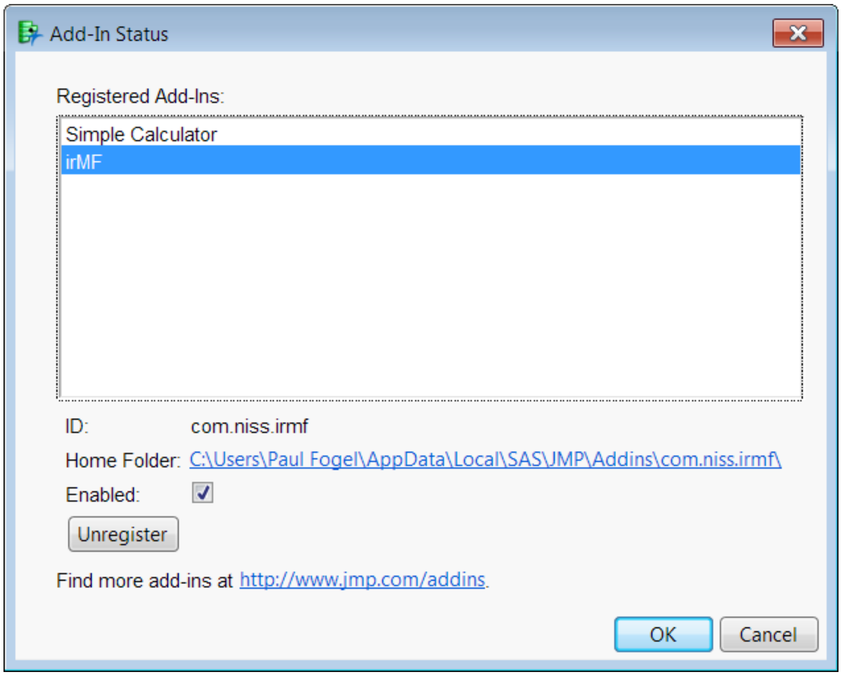

Select irMF add-in and click the button Unregister.

1.4 Professional versus Restricted mode

Certain tabs, functionalities and irMF `Table Subset Management` are inactivated in restricted mode, which occurs after license has expired or when downloading from the NISS site.

Please contact authors in order to receive the "Pro" unlimited version.

2 Data file format

The following data file format must be strictly followed:

Column 1: sample label (**character type only**).

Column 2: group label, leave it empty if no group information available (**character type only**).

Additional group columns may appear immediately after column 2 (**character type only**). To activate a specific group column – see next section on subset selection below – or permute this column with the second column.

Other columns: variables/predictors **must be numeric**. Any non-numeric variable will be ignored.

3 SUBSET SELECTION

In the title of the irMF dialog appears the name of the current data table name. Right below, the button Table Subset Management allows for activating a subset.

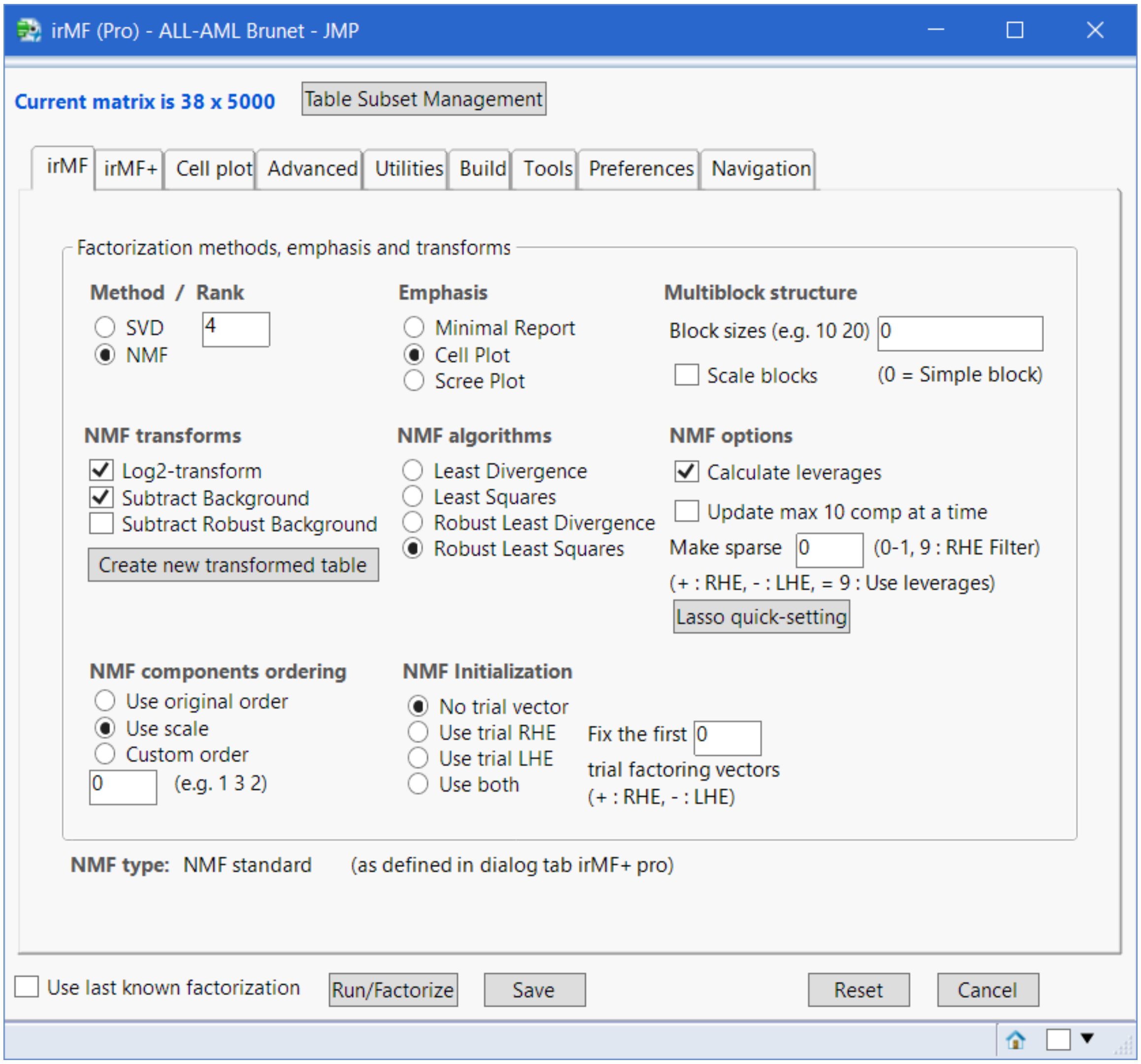

of the table. The subset is automatically defined by the current rows and / or columns selection in the table. Thus, rows or columns should be first selected before clicking on the `Table Subset Management` button in order to define a new subset. Once clicked on, a new dialog is displayed, offering the option to define a new subset from the active selection or to activate an existing subset.

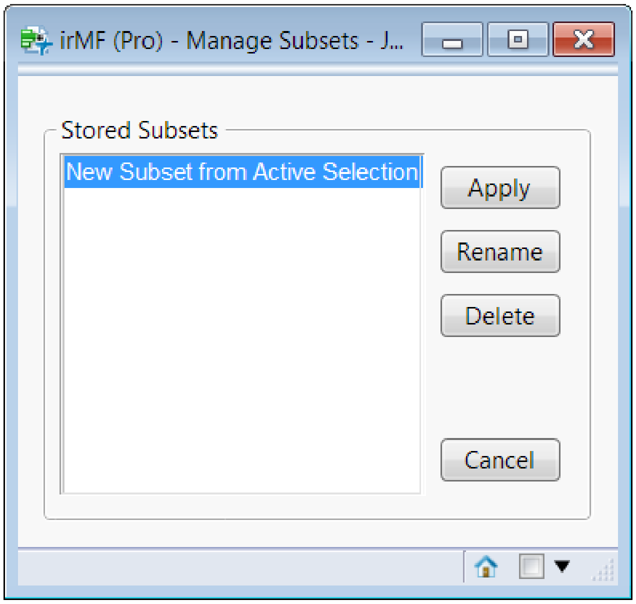

Click on the `Apply` button to generate the new subset. irMF will request the subset name and will store the corresponding subset definition in the table itself (under the script table named `StoredSubsets`). For instance, if the name 'Subset example' was given, it will be displayed in the list of stored subsets next time the `Table Subset Management` button is clicked on, along with the numbers of selected rows and columns (= 0 if no rows or columns were selected, meaning that all rows or columns will be used in the analysis).

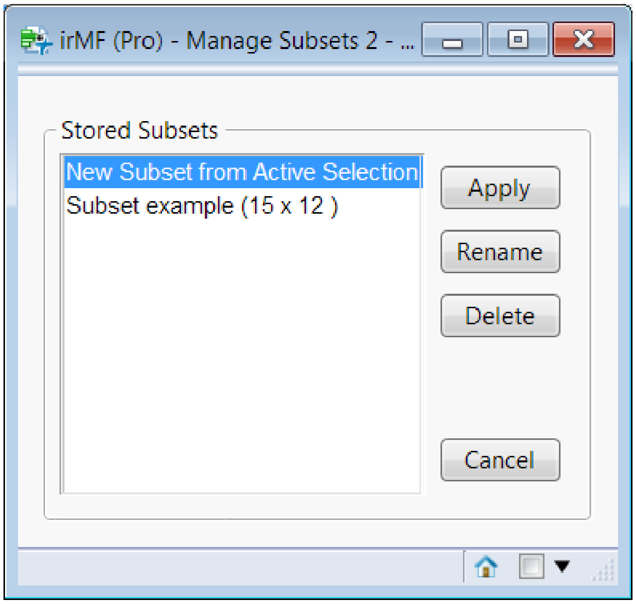

To apply the stored subset, select it and click on the `Apply` button. The name of the subset will appear on the main irMF dialog next to the name of the table:

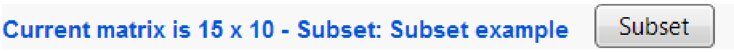

Subsets can be renamed or deleted.

Note: All irMF options applied under a given subset will be specific to the subset itself. These options will be recalled each time the subset is activated.

Note: irMF will automatically apply the last used subset unless the selection of rows or columns has been changed. To disable the subset and work with the complete dataset, open the subset dialog and click on the `Cancel` button.

Tip: If no rows and columns are selected, the entire dataset will be used in the analysis. Still, it is possible to save the entire table as a particular subset under a given name. This is useful to remember different irMF configurations that can be applied on the same dataset.

4 IRMF MAIN DIALOG TAB

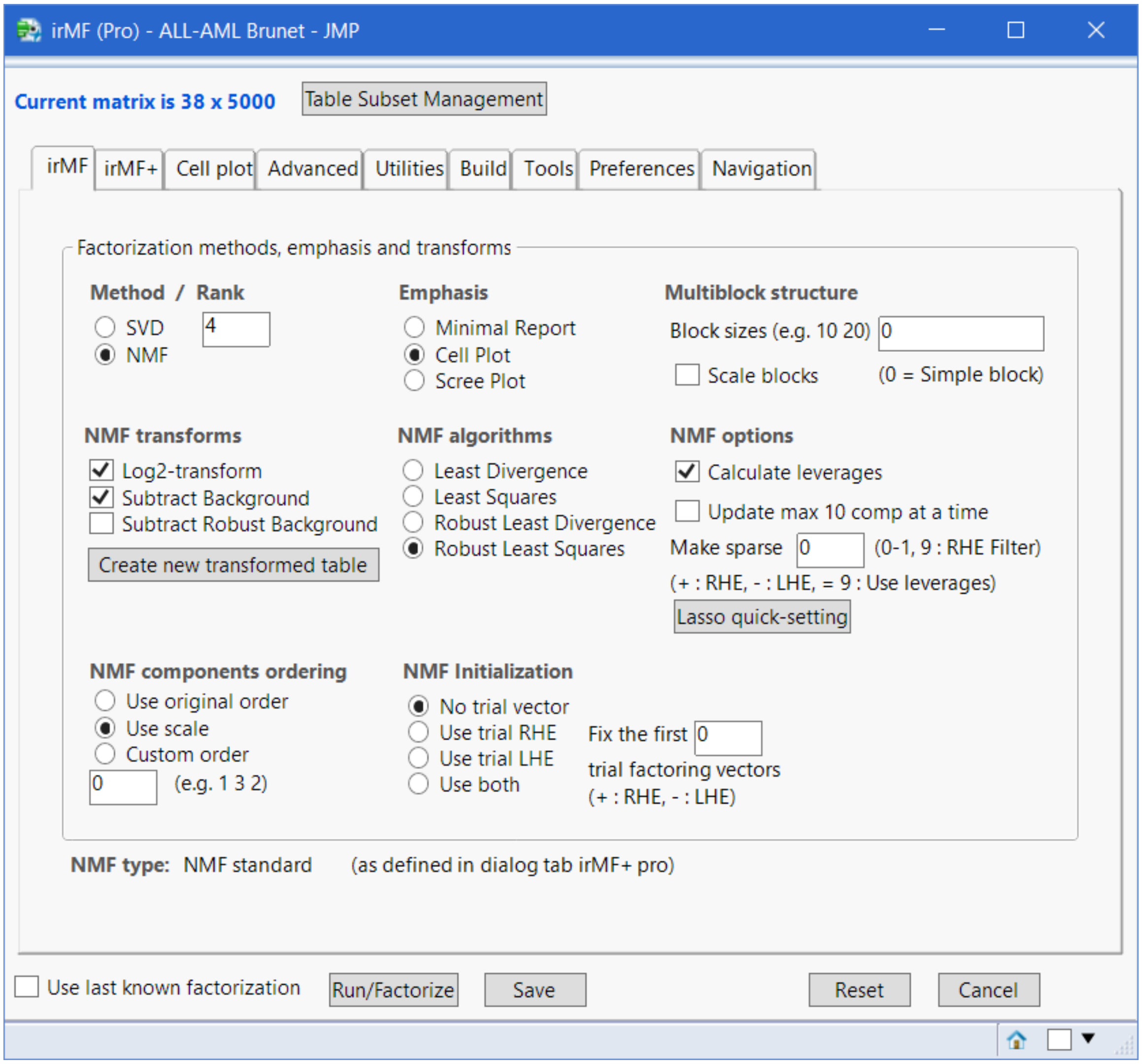

`Factorization methods`: irMF provides two types of matrix factorization: Singular Value Decomposition (SVD) and Non-negative Matrix Factorization (NMF). Note that only SVD accepts matrices with missing cells. Robust SVD is also implemented, following Liu & al (2003). It is possible to apply robust SVD (rSVD) and clean up data (see Build tab) by replacing missing cells and outliers by rSVD model-fitted values prior to running standard NMF.

`Rank`: The number of components to be used in the factorization model must be chosen. Different types of scree plots can be used to help you choosing this number.

`Emphasis`: Minimal Report (factorization model only), Cell Plots or Scree Plots. Additional options for Cell Plot can be found in the `Cell plot` tab. Additional options for the Scree plot can be found in the `Advanced` tab.

`Multiblock NMF`: If the `Block sizes` field is filled to describe a block structure in the columns, then NMF cell plots are organized by the defined blocks.

Tip: By convention, a negative block size will tell irMF to reverse colors in the cell plot for this particular block. Useful when the matrix is split into positive and negative parts (see `Build` tab section).

`Scale blocks:` Check this option to give equal weight to each block, even if one has many more columns than others.

NMF scree plots

Volume

We introduce a novel complementary test that takes advantage of the non-orthogonality of NMF factoring vectors. For a number of components *r*, we calculate the volume of a matrix **Z** having *r* columns $\hat{X}_k, 1 \leq k \leq r$, where $\hat{X}_k$ is the approximation to **X** obtained with *k* components, reshaped into a column vector and normalized. To calculate the volume, we take the determinant of **Z'Z**. irMF provides a simultaneous plot of:

(i) The volume achieved with models corresponding to 1, 2, ..., *r* components (max volume = 1 means that components are orthogonal). Volume decrease indicates that components are correlated.

(ii) Mean square residuals (MSR), normalized to have max MSR = 1.

The volume criterion can evolve in different ways, depending on the nature of underlying mechanisms:

- Independent mechanisms (e.g. data is a mixture of independent sources): Volume remains stable as long as the number of components is smaller than the number of mechanisms. The volume shows a sharp decrease once the number of components exceeds the number of mechanisms.
- Dependent mechanisms (e.g. expression profiles that share common pathways): Components being associated with mechanisms are correlated. Therefore, the volume decreases substantially until all existing mechanisms can be associated with their own component. Volume stops decreasing (or keeps only decreasing slightly) when extra components, which explain noise rather than signal are added, due to the inherent orthogonality of added noise.

Stability and specificity

The stochastic nature of the *robust* version of the NMF algorithm is described in section 4.1.1, options for NMF. This algorithm provides a means to assess whether a given rank *r* provides a meaningful decomposition of the data. Each sample being clustered with a given frequency into a particular cluster, the mean frequency over all samples is an indicator of the stability of the clustering: the higher the mean frequency, the more consistent the clustering across all runs. Similarly, an indicator can be constructed regarding the stability of the clustering of variables. Both indicators tend to become unstable when *r* becomes too high. The overall specificity of the left or right factoring is an indicator of the level of discrimination between associated clusters.

Note: NMF scree plots are time-consuming. However, in datasets with numerous redundant variables, like microarrays, experience shows that very similar NMF scree plots can be obtained from a smaller subset of variables sampled at random.

Note: Clusters of rows or columns are associated with each component. Too high a number of components may lead to empty clusters (i.e. the resulting classification function does not cluster any row or column into this cluster).

Options that are specific to the chosen factorization method are displayed. Options that are specific to the un-chosen factorization method are hidden.

! In the following, we assume that NMF option has been selected

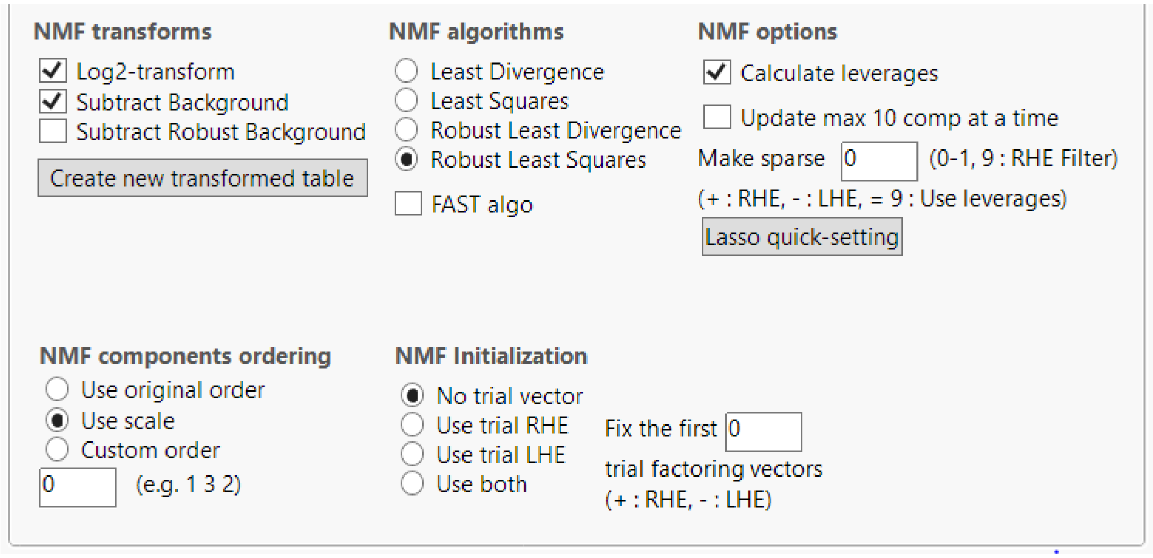

`NMF transforms`: `Subtract Backgound` removes the lowest value from each column so all columns become positive and the smallest value in each column equals 0. Both options are recommended if the dataset is log-distributed and Least Square method is used. The option `Subtract Robust Background` prevents outliers from distorting the profile of column minimums, by projecting the original profile of column minimums onto the profile of column 10%-quantiles. Using the latter option can yield negative values in the transformed matrix, which are all replaced by 0 values.

`Create transformed table`: Use this option to output a table with transformed values.

`NMF algorithms`: Choose between Least divergence, Least squares, Robust Least divergence or Robust Least squares. The robust approach is described below. We recommend using Least squares, unless the data is Poisson distributed, in which case Least divergence is recommended (although much slower than Least Squares).

`FAST algo`: Useful with matrices with large numbers of rows and columns, e.g. SNP data. NMF Fast algo expects that tables generated by a truncated SVD are already open (see detailed description in dedicated document).

NMF options (irMF PRO)

`Calculate leverages`:

NMF squared elements of the columns of **W** and **H** are typically constrained to sum to unity, as a convenient way of eliminating the degeneracy associated with the invariance of **WH** under the transformation **W** →**W**Λ, **H** → Λ^{-1} **H**, where Λ is a diagonal matrix defining a particular scaling system. It should be noted that sum to unity constraint is arbitrary.

A simulation example will illustrate this scaling issue. In the NMF score plot (Figure A1), each sample is projected onto a plan. Recall that coordinates (*x*, *y*) guarantee that the weighted sum: S = $xH_1 + yH_2$ closely approximates the real sample. Two scaling systems were applied to determine (*x*, *y*): One scaling system ensures that the elements of the feature vectors H_1 and H_2 vary within a “realistic” range – similar to the original features (see Figure below). Thus, (*x*, *y*) reflect the proximity of each sample to either feature vector. Samples marked with a circle (5 red circles, Figure a) are closer to H_2 and samples marked with a cross are closer to H_1 (15 blue crosses, Figure a). The second scaling system is determined by the square root of the sum of squares of each column vector of **W**. Thus, it is agnostic to the range of values taken by original features. If the latter scaling system were to be used for clustering samples – instead of the first “realistic” scaling system – additional samples (Figure b, 3 red crosses) would appear wrongly clustered with the H_2 cluster (since *y* > *x* in this particular scaling system). If the distance to each feature vector is now defined by the Euclidian distance to the extremity of each axis, the clustering remains error free in either scaling system (Figures a and b, blue lines).

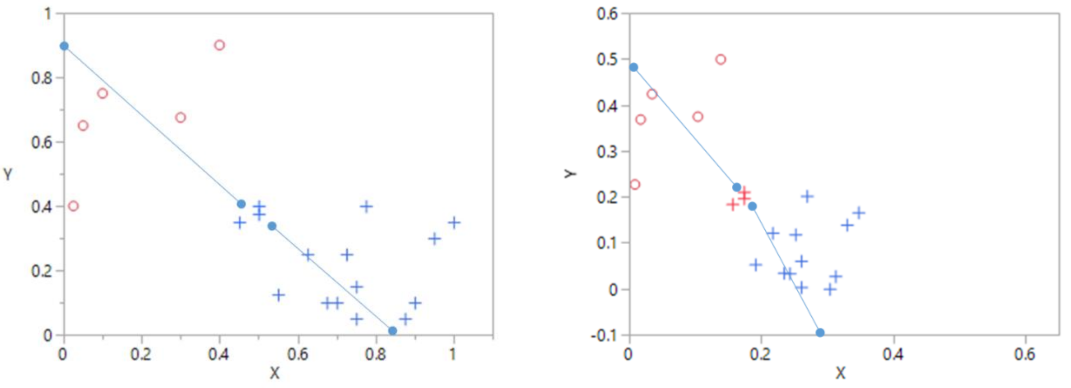

(**a**) (**b**)

(**a**) Distance wrt original scale; (**b**) Distance wrt L2 scale

This distance, which appears to be robust to the chosen scaling system, can be easily extended to more than two component vectors. For each observation *i*, the distance to each feature vector *q* is defined by:

$$distance(i,q) = \left(\max(\mathbf{W}(.,q)) - \mathbf{W}(i,q)\right)^{\mathbf{2}} + \sum_{r\neq q}^{k} \mathbf{W}(i,r)^2$$

The *leverage* can be directly derived from the distance:

$$leverage(i,q) = 10^{-\frac{distance(i,q)}{2\cdot mean(distance(.,q))}}$$

By construction, *leverages* are well correlated with factor components **W** or **H** and range between 0 and 1, however they are little affected by the chosen scaling system. Thus, the comparison between leverages allows for a reliable clustering.

`Update max 10 comp at a time:` This option is useful whenever the requested number of components is high and the computation time becomes too long. When activated, only 10 components chosen at random are updated at each iteration of the alternate least squares algorithm.

`Make sparse:` A sparse vector has many elements at or near zero. Sparse factoring vectors are potentially useful: sparse vectors are easier to interpret and it is unlikely that all predictors are involved in a specific mechanism. During the updating process, for each factoring vector, a lasso-type approach is used. The threshold indicates the requested proportion of samples or variables (depending on the sign of the threshold) with 0 element on any of the components. If set to 0, the sparseness constraint is inactive. The coded value 9 does not use Lasso. For more details, please contact the authors.

`Lasso quick settings:` By clicking on this button, several parameters are simultaneously changed: The sparseness threshold is set to 0.5 and the number of fixed left factoring vectors is set to the rank of the factorization. This option is useful for variable selection: Once the full model has been estimated, Lasso will allow for removing as many variables as possible, while ensuring that the left factoring vectors remain very similar, whether based on all variables or only selected variables. The table subset window will pop up automatically to allow for creating the subset of lasso-selected variables.

`Standard settings:` By clicking on `Standard settings` (same button as above, with replaced label), it is then possible to run again NMF and estimate new patterns (left factoring vectors) based on selected variables. And eventually check that new and old patterns are well correlated, meaning that excluded variables did not result in loss of information.

`Components ordering`:

NMF does not guarantee that factoring vectors are found in decreasing order of their respective scale. Check the option `Keep vectors in original order` if you want factoring vectors in the order of computation. Check the *option* `Use scale` if you want factoring vectors to be ordered by descending scale. Check the option `Custom order` if you want factoring vectors to appear in a specified order (e.g. if you want one particular cluster to appear next to another one).

> Note: The Custom order option can be used along with the `Use last known factorization` option in the following way: (i) Run NMF with scale ordering and with `Use last known factorization` option unchecked (ii) Based on the output, enter the most appropriate components ordering, e.g. 2 1 4 3 (iii) Re-run NMF this time with `Use last known factorization` option checked (this option will be automatically checked after entering a custom ordering).

`NMF initialization` **(irMF PRO)**

By “NMF initialization”, irMF means the process of running NMF twice, the first time to initialize the model (using `Options for NMF` as described above), and the second time to impose some additional constraints on the factorization.

`Trial factoring vectors`: irMF allows the user to initialize the factoring vectors. Run first irMF with no trial vectors. Examine the factoring vectors output table Right and/or Left factoring vectors, make all desired changes and choose between one of the options `Use trial RHE`, `Use trial LHE` or `Use both` (RHE/LHE stand for Right/Left Hand factoring vEctors). If there are specific “known” factoring vectors, they can be fixed provided that they appear first in the factoring vectors output table. In this case, the sign convention determines whether left or right vectors should be fixed when both trial LHE and RHE are used. If necessary permute columns of the table of factoring vectors to have fixed factoring vectors first in the table. Only non-fixed trial vectors will be updated. irMF assumes that trial factoring vectors are equally important so scales are all initialized to one before the fitting process starts.

> Note: If some of the trial vectors are fixed, the option `Keep factoring vectors in original order` (see below) is automatically turned on.
>
> Note: Another strategy is to take the effects of “known” factoring vectors out of the matrix. The residual matrix can be obtained from the `Build` tab (see below) and then be used as input to irMF to further analyze the data set.
>
> **! In the following, we assume that SVD option has been selected**

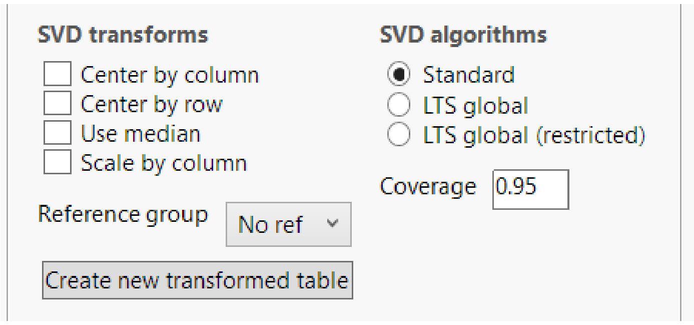

`SVD transforms:` These options correspond to standard normalization procedures. The default is no centering. If option `By row` or `By column` is checked, then the default is to use the mean of rows or columns. The median is used instead if the option is checked. A particular group can be selected in order to normalize columns. In such case, the mean or median of this group will be subtracted.

`Build transformed table`: Use this option to output a table with transformed values.

`SVD algorithms:` Standard and two robust SVD options are currently implemented, following Liu & al (2003). Standard SVD is computed using alternating least squares. Least trimmed squares replaces the least squares regression by using the k data points that have the lowest sum of squared residuals.

! The following boxes are displayed at the bottom of the dialog if any of the tabs *irMF*, *irMF+*, *Cell plot*, *Advanced* or *Preferences* is activated.

The option `Use last known factorization` is accessible from all tabs: Last factorization results can be used to bypass calculations and apply changes in output options. This option is particularly useful with large datasets.

`Run/Factorize:` Run irMF with selected options.

`Save`: Save the table with current irMF configuration parameters.

`Cancel:` Disregard selected options and close irMF dialog.

`Reset`: Click this button to clear the irMF environment (tables and specific namespace variables). This option is particularly useful to reinitialize NMF models that are stored in memory.

5 OTHER DIALOG TABS

5.1 IRMF+

The irMF+ tab has two panels: `Options for robust NMF` and `Options for NMF variants`.

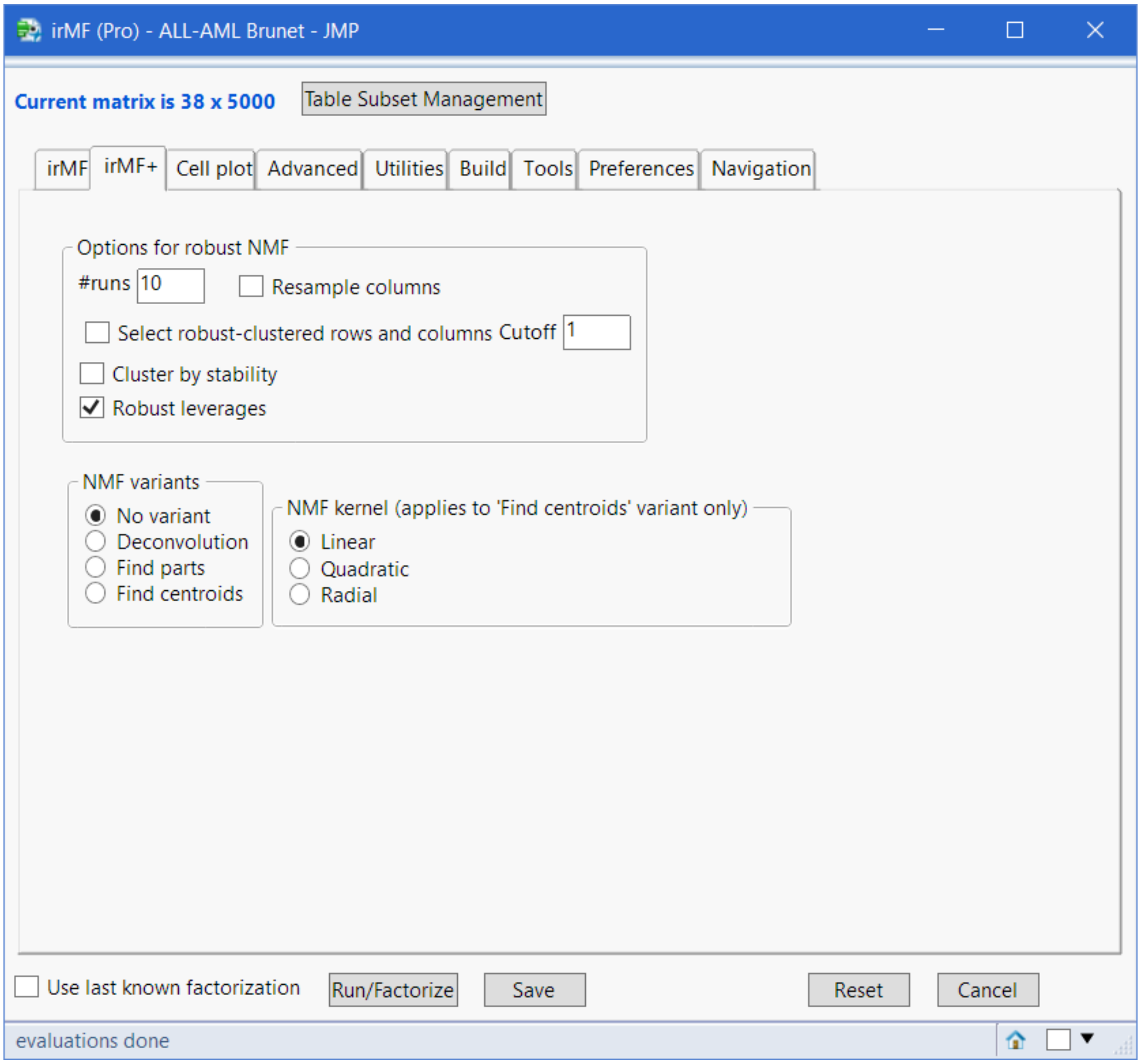

5.1.1 Robust NMF

The stochastic nature of most clustering algorithms has been shown to be rather useful in providing methods for evaluating the consistency and robustness of their performance (Devarajan, 2008). The central idea is to perform numerous runs of the algorithm starting from different random initializations. On each run, the algorithm groups the samples into clusters, allowing for the calculation of the frequency at which two different samples fall into the same cluster. In contrast to random initialization, we first calculate a pseudo-unique NMF solution based on SVD initialization, which is itself unique (Boutsisdis, 2007). In order to evaluate the robustness of the clusters, samples are bootstrapped along with left factoring vectors and right factoring vectors are re-estimated on each run. Since different right factoring vectors give rise to different clusters of variables, the frequency at which a variable falls into a particular cluster can be calculated, the highest frequency determining the most reliable, robust cluster for any variable. The right factoring vectors, which are obtained on each run of the bootstrap, can be used in the reverse way to re-estimate left factoring vectors, which in turn determine sample clusters. Similarly, the frequency at which a sample falls into a particular cluster can be calculated, the highest frequency determining the most reliable cluster for any sample. Note

that other algorithms using random initialization do not guarantee any consistency in the ordering of the clusters, i.e. the same cluster can appear as number 1 or 3 depending on the initialization. As a consequence, it is not possible to assess directly the frequency at which a sample falls into a particular cluster, as we do here. Thus the use of more complex measures, such as the cophenetic correlation, to assess whether sample pairs tend to be consistently clustered together or not.

All options below relate to the robust algorithm:

`#runs`: Enter here the number of bootstrap runs to be used by the robust NMF algorithm.

`Resample columns`: Check this option if you want to swap roles between columns and rows during the robust estimation process. This option is disabled if Multiblock NMF is used or trial factorial vectors are used.

`Select robust clustered rows and columns`: Rows or columns which consistent clustering frequency is higher than the entered threshold will be automatically selected.

`Cluster by stability`: Rows or columns are by default clustered with respect to their respective elements within left or right factoring vectors, or corresponding leverage – if the option `Calculate leverages` is activated, i.e. the component with the largest element or leverage defines the row or column clustering. It is possible instead to use the stability frequencies, i.e. choose the component with largest clustering frequency.

`Robust leverages`: The distance to the *q*th feature vector (see section on the calculation of leverages) is determined by $\max(\mathrm{W}(.,q))$, which can be obviously affected by outliers. It is therefore important to calculate a *robust* maximum for each component *q*, which would take into account the sample specificity (Section 2.2.3) along with its weight on the *q*th component. The following iterative algorithm allows for estimating such robust maximum:

1. Initialize the robust maxima by the maximum of each component
2. For each vector component *q*:
    a. Calculate the probabilities and specificity of each row (section 2.2.3)
    b. Force the specificity to 0 if any of these two conditions holds:
       $p(i, q) < 1 / k$
       $\mathbf{W}(i, q) <$ Quantile 95% ($\mathbf{W}(., q)$)
3. Update Robust Max(*q*) by the mean of all $\mathbf{W}(i, q) >$ Quantile 95% ($\mathbf{W}(., q)$), where the mean is weighted by the row specificities.
4. Replace all $\mathbf{W}(i, q) >$ Robust Max(*q*) by Robust Max(*q*)
5. Repeat 2. until convergence

Back to the simulation example given in the section `Calculate leverages`, the maximum appears above the horizontal blue line representing the robust maximum in the histograms of each column vector. Note that on the second component, the maximum was also identified as an outlier by the standard criterion based on the interquartile distance.

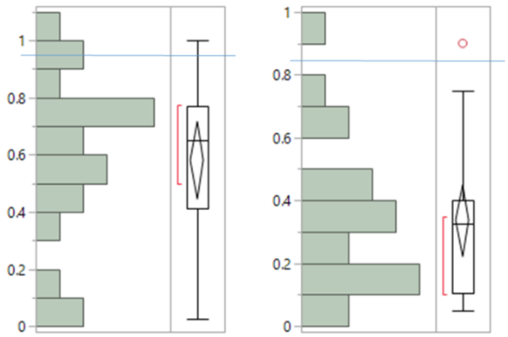

Estimation of a robust max on each component vector (blue line on top of each histogram

5.1.2 NMF variants (irMF PRO)

Version 7.0.0 introduces new variants of NMF along the lines of Ding, C.H.Q.; Tao, L.; Jordan, M.I. Convex and Semi-Nonnegative Matrix Factorizations. *IEEE Transactions on Pattern Analysis and Machine Intelligence archive* **2010**, *Volume* 32, no. 1 pp. 44–55.

More details are given in a separate document.

5.2 CELL PLOT

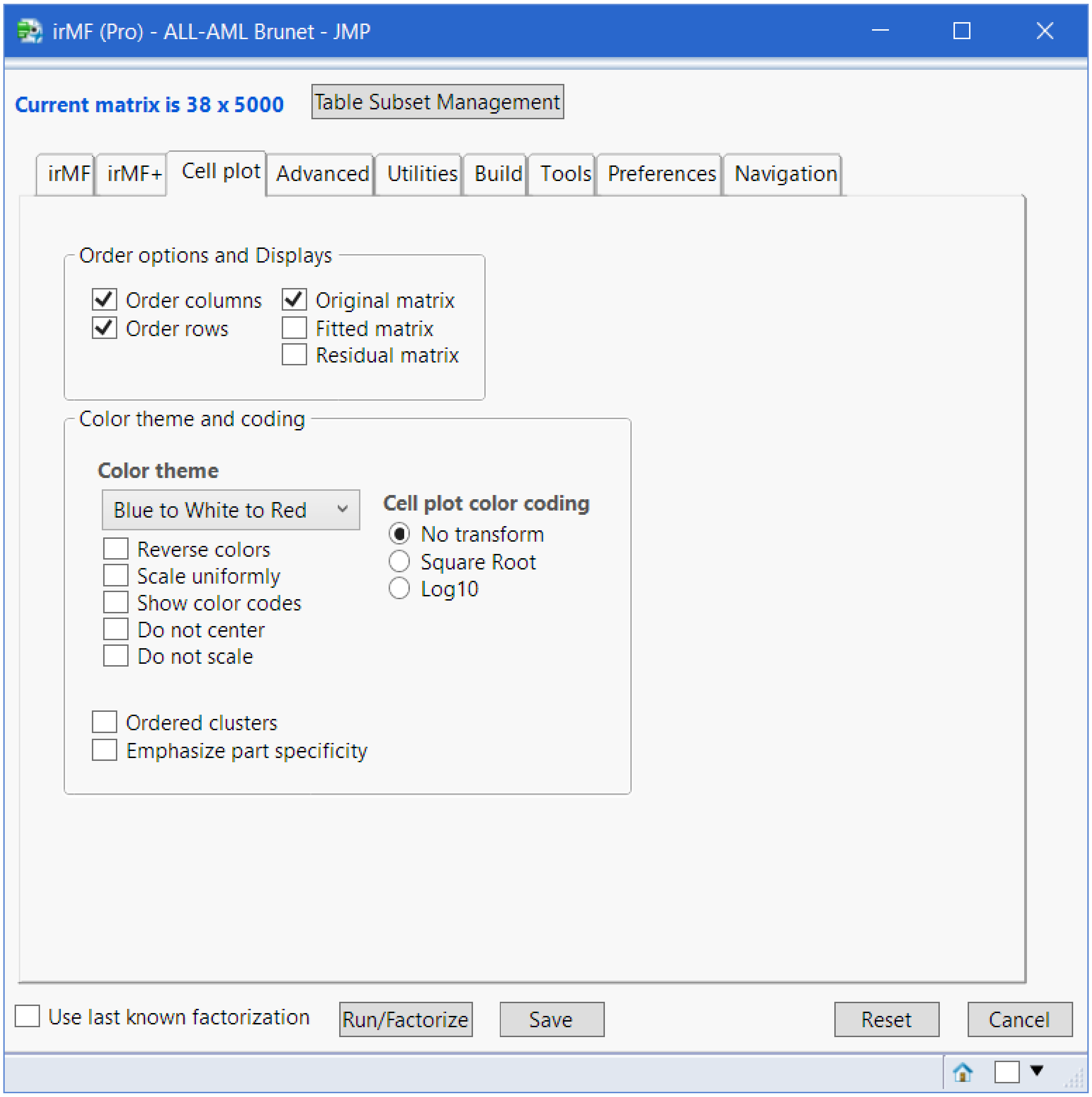

`Order options and Displays`: Rows and columns of the original matrix can be reordered by decreasing values of the elements of the right and/or left factoring vectors. Thus there are as many possible permutation matrices as there are components in the model. These permutations may apply to original, fitted, or residual matrices. The permuted matrices can be displayed in the form of a cell plot (heat map). NMF bi-clustering provides a single combined cell: rows and columns are reordered by decreasing values of the elements of the right and/or left factoring vectors associated with each cluster. The ordering of clusters within combined cell plot follows the ordering of components (see above the components ordering section). Stability frequencies and leverages are represented in additional cell plots on the right and bottom side of the combined cell plot, to help determine the beginning and end of each cluster.

`Color theme:` Apart from listed color themes, there are options for not centering and/or not scaling columns (the default is to center and scale columns). These options are useful when working with split matrices (concatenated positive and negative parts of the original matrix).

`Color coding`: The color coding is based on the standard deviation of concatenated columns. If the cell plots appear uniformly green or red, then try using log-normal or square-root transform. Note that the transform applies only to the way colors will be coded in the cell plot. The matrix factorization itself applies to original or normalized data if these options are checked in the main dialog tab.

> Note: This option is disabled if at least one of the NMF normalization scheme (irMF tab) is checked.

The cluster plot is specific to NMF. NMF can be used to cluster a data set. Consider the left factoring vectors. Each row is assigned the cluster number of the left component with the highest element. Likewise the columns can be assigned the number of the right component with the highest element so the matrix can be bi-clustered. The matrix is permuted in the order of decreasing values of the elements of the factoring vectors, cluster by cluster.

> Note: In the special case of two clusters, ascending order is used to order the elements of the second factoring vector. This way, a continuous map is created in both directions, e.g. on the X-axis going progressively from most up- to most down-regulated genes.

`Ordered clusters:` Check this option if clusters have been ordered in order to achieve a continuous change in patterns. The ordering of rows and columns within each cluster will be affected. However, the clustering itself remains unchanged.

`Emphasize part specificity:` This option is depreciated.

5.3 ADVANCED

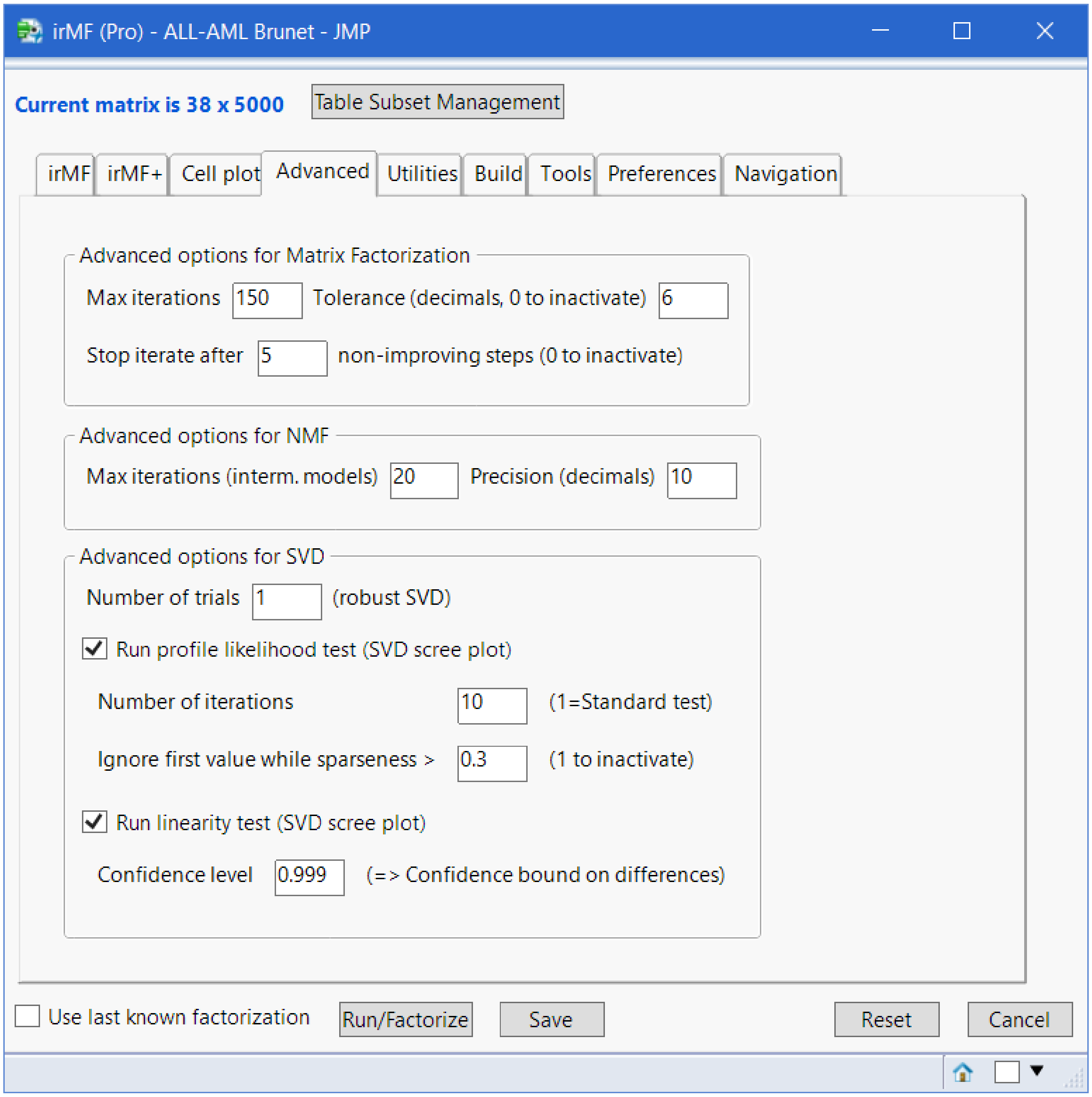

`Advanced options for Matrix Factorization:`

- **`Max iterations`**: 200 are recommended, although convergence is generally ensured within 100 iterations. If least divergence is used, the actual max number of Li-Seung iterations is multiplied by 10, since convergence is known to be much slower.

- **`Tolerance`**: This parameter controls the convergence; we stop when the Mean Square error does not change more than the tolerance level.

`Advanced options for NMF:`

- **`Max iterations (intermediate models):`** When the least square method is used, this parameter defines the number of Lee-Seung preliminary iterations that are necessary to initialize projected gradient.

- `Precision`: When the least divergence method is used, 0 values will be replaced by a small positive value 1e-**Precision**. Replacing 0 values is important to prevent calculation underflow.

`Advanced options for SVD:`

- `Number of trials`: Several trials can be performed when using the robust algorithm, which is initialized in a stochastic way. The best solution will be returned. Our experience is that using only one trial is generally enough to get a solution close to optimal.

- `Profile likelihood and linearity tests`: In developing an approximation to the matrix X, the number of right and left factoring vectors, k, needs to be specified or determined.

  The profile likelihood test for finding the optimal number of components, k, is adapted from Zhu and Ghodsi (2006). One assumes that eigen values follow a mixture distribution of two normal distributed populations; one group corresponding to real components (the larger eigen values) and the other noise (the smaller eigen values). We calculate the profile likelihood for any hypothesis of k significant components under the assumption that both populations have same standard deviation. It makes sense to select the hypothesis number that has the highest profile likelihood.

  The linearity test is performed on the differences between consecutive eigen values, which should be constant for those components which are noise. We compute an upper confidence bound for those differences. The linearity test plot should be read from right (smallest component) to left (largest component): the first time the difference between consecutive eigen values exceeds the upper bound line can be taken as the optimal number of components.

5.4 UTILITIES

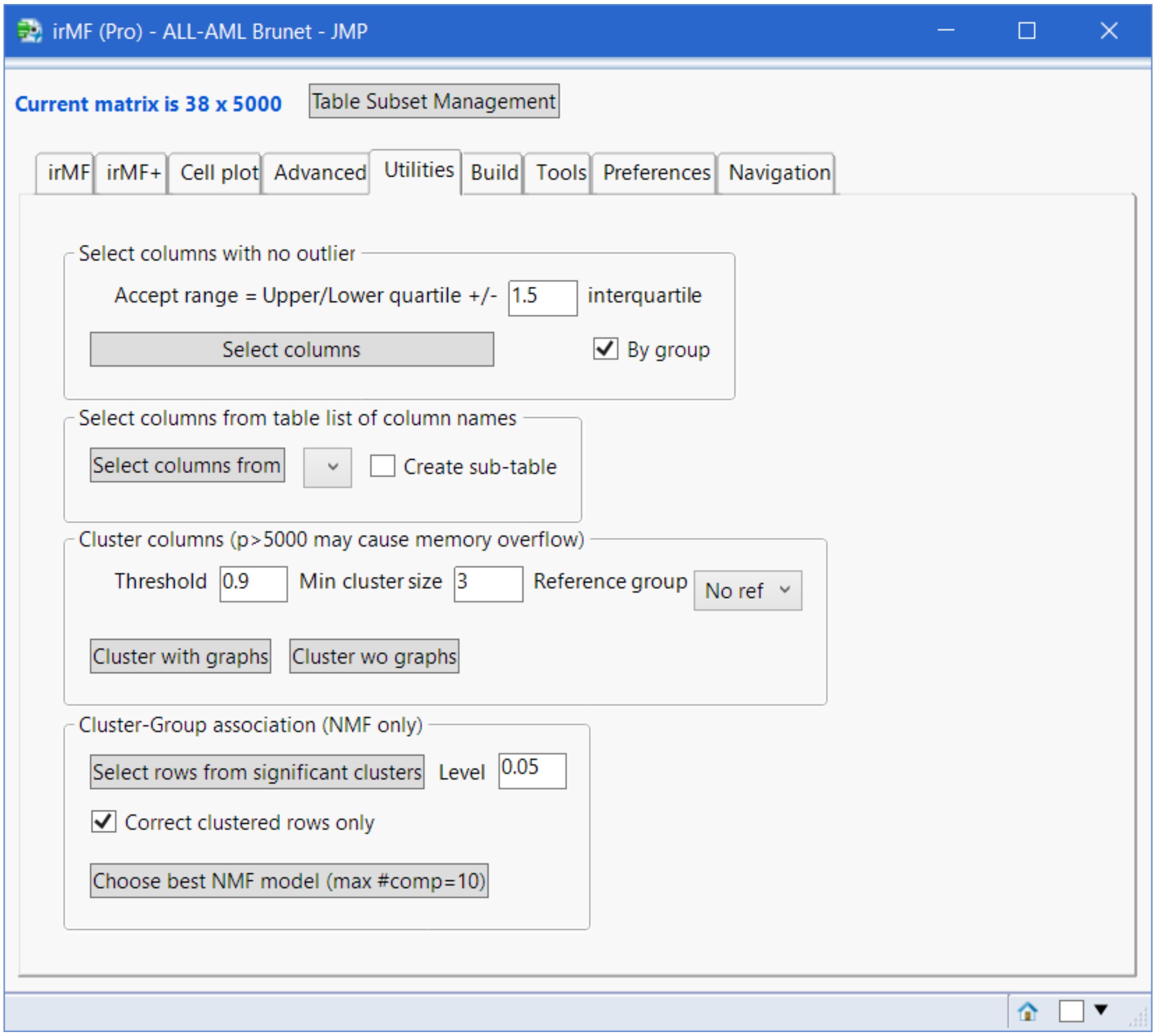

For the JMP active data set, utilities are provided to help generate tables for future use with irMF or other platforms:

`Select columns with no outlier`: Removing columns with univariate outliers can be useful for NMF pre-processing or if one wants to reduce the number of columns. If there are treatment groups, it makes sense to check for outliers within each group.

`Select columns from list of column names`: Column names can be selected from any table to generate subset that will contain corresponding columns in the irMF table.

`Cluster columns`: Based on a simple "Leader" clustering algorithm proposed by J.Liu (personal communication), in which pairwise correlations between columns are used:
Step 0: select or filter the variables list
Step 1: Starting with no clusters, calculate all the pairwise correlations and select the pair of variables with the highest correlation.
Step 2: Find the strongest correlation in the unclustered columns to the leader and form a new cluster for those two correlated columns.

Step 3: From those unclustered columns find the column that has the best average correlation with the cluster formed in step 2. If the average correlation is larger than a user-specified cutoff (e.g. absolute correlation is larger than 0.9), add the column to the cluster; otherwise go to step 2 and restart a new search.
Step 4: Repeat procedure 2 and 3 until no pairwise correlation is found to be larger than the cutoff.

This algorithm can be used to exclude columns that do not fit in any of the found clusters (cluster 0). To do that, use the "Select columns" option as described above and select the table "(irMF) Column clusters" which is created by the clustering program.

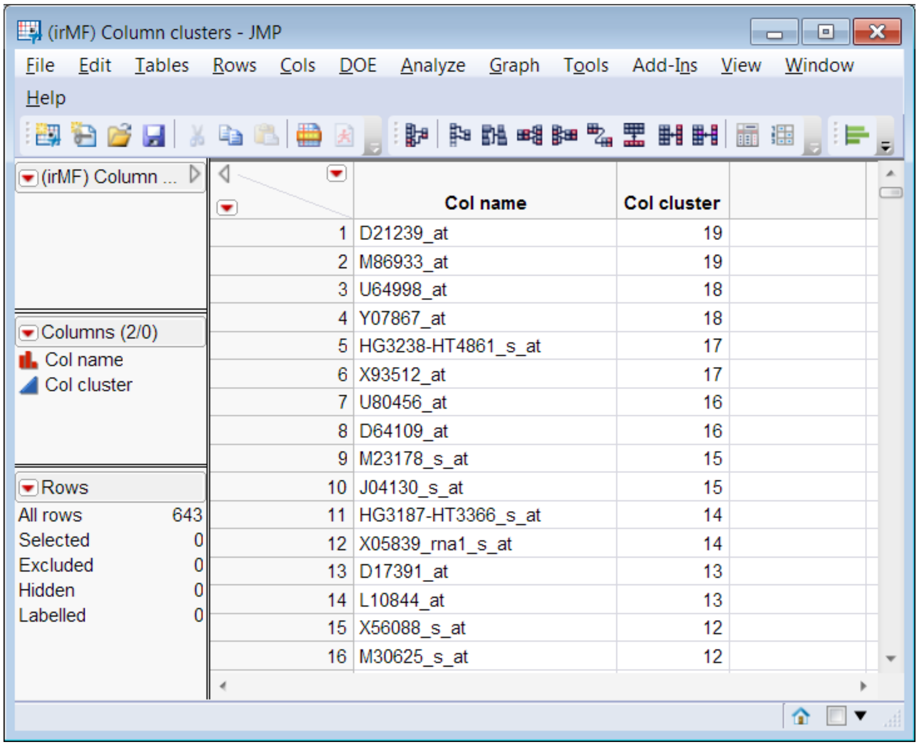

Select cells from the "Col name" column which adjacent cells in the "Col cluster" column are not zero.

New!

- Cluster columns with respect to a reference group
- Maximize contrast in entropy between the reference group (low entropy) and the other group (max 2 groups only)

`Select rows from significant clusters`: Recall that for each cluster, the cluster sample with the highest LHE element points to the associated experimental group. We use the hypergeometric distribution to test this association. If significant, rows from the cluster are selected.

`Correct clustered rows only`: Check this box if you want only correct clustered rows within significant clusters to be selected.

`Choose best NMF model`: In order to choose an appropriate model, one strategy is to run NMF with 2, 3, 4, etc. components and optimize the association between found NMF clusters and groups. irMF performs permutation runs to see how “lucky” the found associations can be. The procedure is following:
First test all the models (up to 10 components accepted). The resulting clustering's are stored automatically into memory. Then let irMF choose the best model according to an association score (described below) and assess the significance of the association over all tested models.

> Note: To start with a fresh series of models, all internally stored NMF models can be erased from memory through the option *Reset* (see irMF main tab)

The internal algorithm is as follows:
For each run:
1) Permute row group labels.
2) For each model, calculate a score associated with the quality of the association.

3) Take the max score across all models and compare with the original max score found without permutation. If larger, then increment a counter.

After 10000 runs, p-value = count/10000

Note: The number of runs can be redefined in the Preferences dialog tab (see below).

One difficulty is in defining an appropriate score to assess the quality of the association that can be compared between different permutation runs and models (there is a risk of model over-fitting, pointing to non-relevant clusters). irMF essentially uses a chi-square score of independence between the rows and columns of a table. Here the rows are the clusters, the columns are the groups, and each cell contains the number of observations that belong to the corresponding group and cluster. The ordering of rows within a cluster is important. For this reason, each count is weighted by the value of corresponding element in left hand vector, giving rise to a *weighted* score.

For each cluster, we add an association significance (based on the hypergeometric distribution) in the cell which corresponds to the main group within cluster.

Group size by cluster & significance of main group:
[using hypergeometric distribution]

Cluster(Size)	ALL_B1	ALL_B2	ALL_T	AML
C 1 (9)	8 [<0.0001*]	0	0	1
C 2 (10)	2	8 [<0.0001*]	0	0
C 3 (8)	0	0	8 [<0.0001*]	0
C 4 (11)	1	0	0	10 [<0.0001*]

Weighted score: 43.14
Global significance using 10000 permutations: 0.0001

Note: NMF produces for each model a global p-value of the association between clusters and groups. This p-value is calculated in a similar way, but is associated with the model being tested, not *all* the models that have been tested on the same dataset.

5.5 BUILD

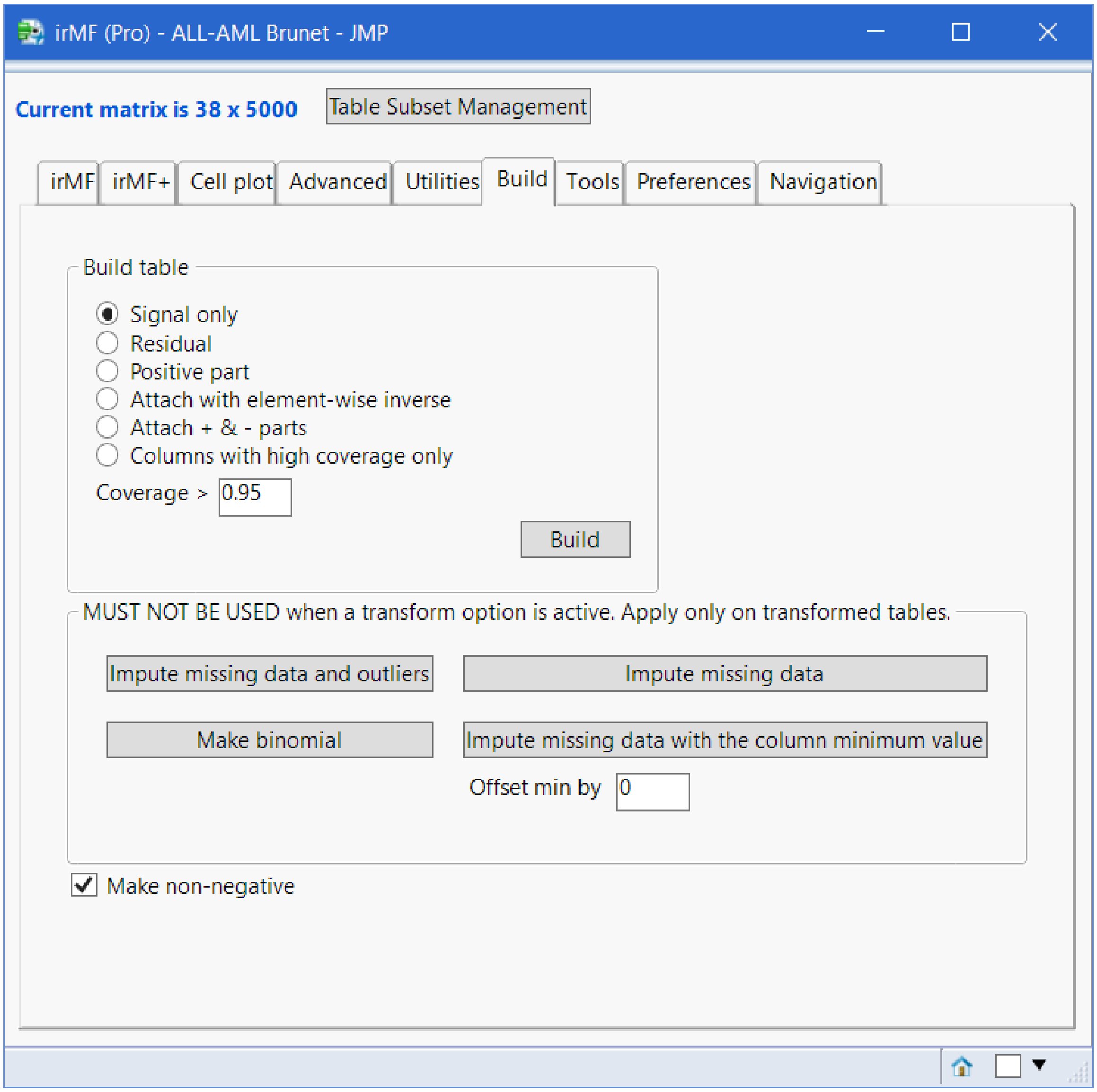

`Signal only`: Use this option to output a table with model-fitted values.

`Residual`: Use this option to output a table with model-residual values.

`Positive part`: Use this option is you choose to ignore negative values, which will be replaced by 0.

`Attach with element-wise inverse`: In micro array experiments we are typically interested in both up and down regulation. NMF focuses on the large positive values. If every element in the matrix is replaced by $1/x_{ij}$ then small values become large. This option can be

used to run multiblock NMF on both tables simultaneously. The next option *Attach + and – parts* is recommended though.

`Attach + and – parts`: This option is useful when a subject effect or the overall mean of each column has been subtracted from the original signal, resulting in positive and negative values. The matrix is then duplicated in positive and negative parts, and negative values are replaced by their absolute value. Use this option to run multiblock NMF on both tables simultaneously.

`Columns with high coverage only`: Use this option to output a table with only columns having less than xx% outlier (as detected by robust SVD) or missing cells.

The following option MUST NOT BE USED when a transform option is active. Apply only on transformed tables!

- **`Impute missing data and outliers / Impute missing data:`** Use these options to impute and replace missing cells and outliers using rSVD model-fitted values. It may be useful to “cure” a matrix prior to run standard NMF.
- **`Make binomial:`** Replace matrix values with 1/0, 1 for all non-missing and 0 for all missing data.
- **`Impute missing data with the column minimum value`**: Use this option to impute and replace missing cells within a column by the column minimum value.

`Make non-negative`: SVD based fitted values are not guaranteed to be non-negative. Check this option to replace negative values by 0 (default).

5.6 TOOLS

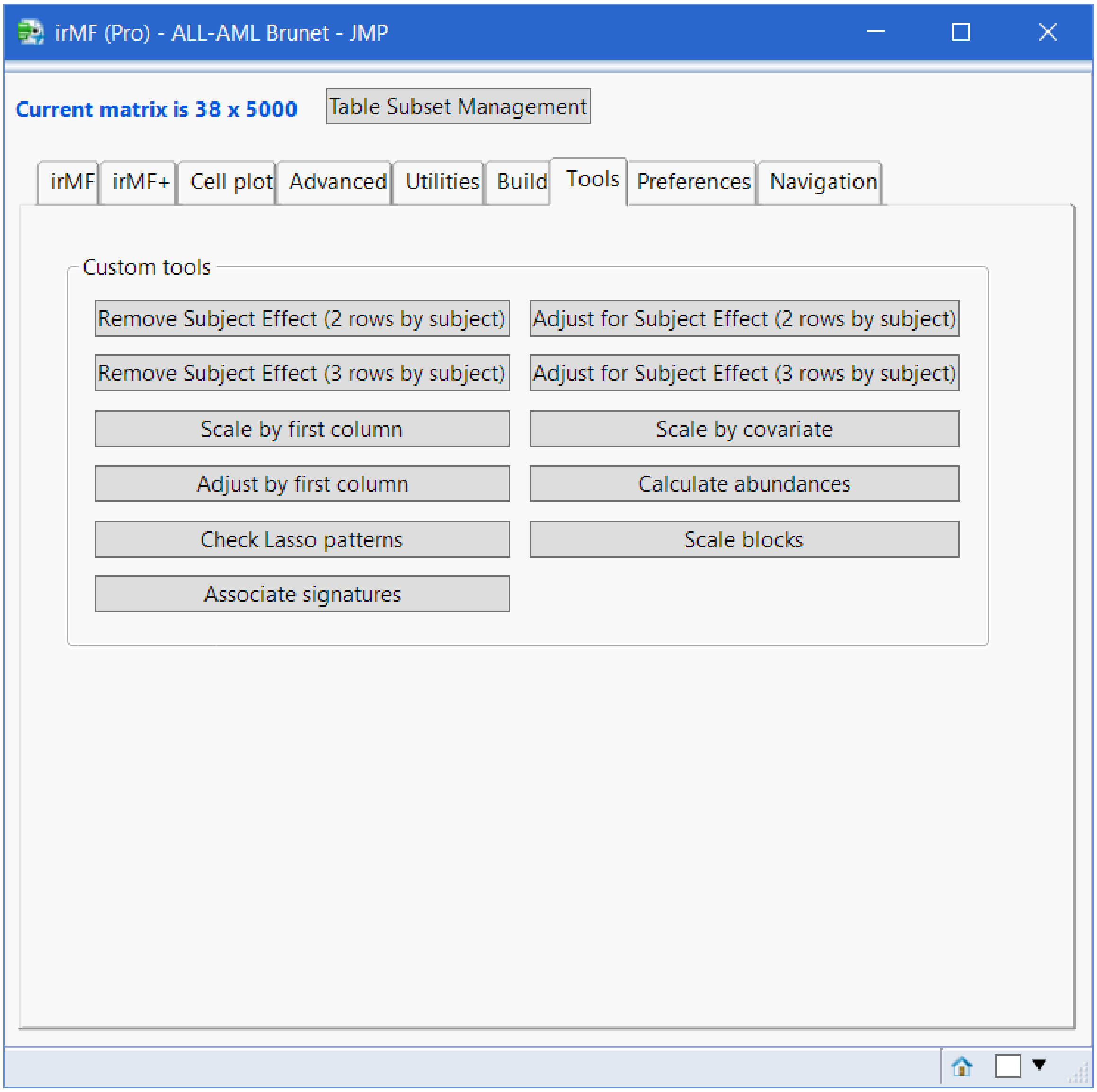

Tools are experimental functionalities that can be easily accessed and modified (the jsl scripts are not encrypted). These tools are still not part of irMF itself and are therefore not described in this document.

5.7 PREFERENCES

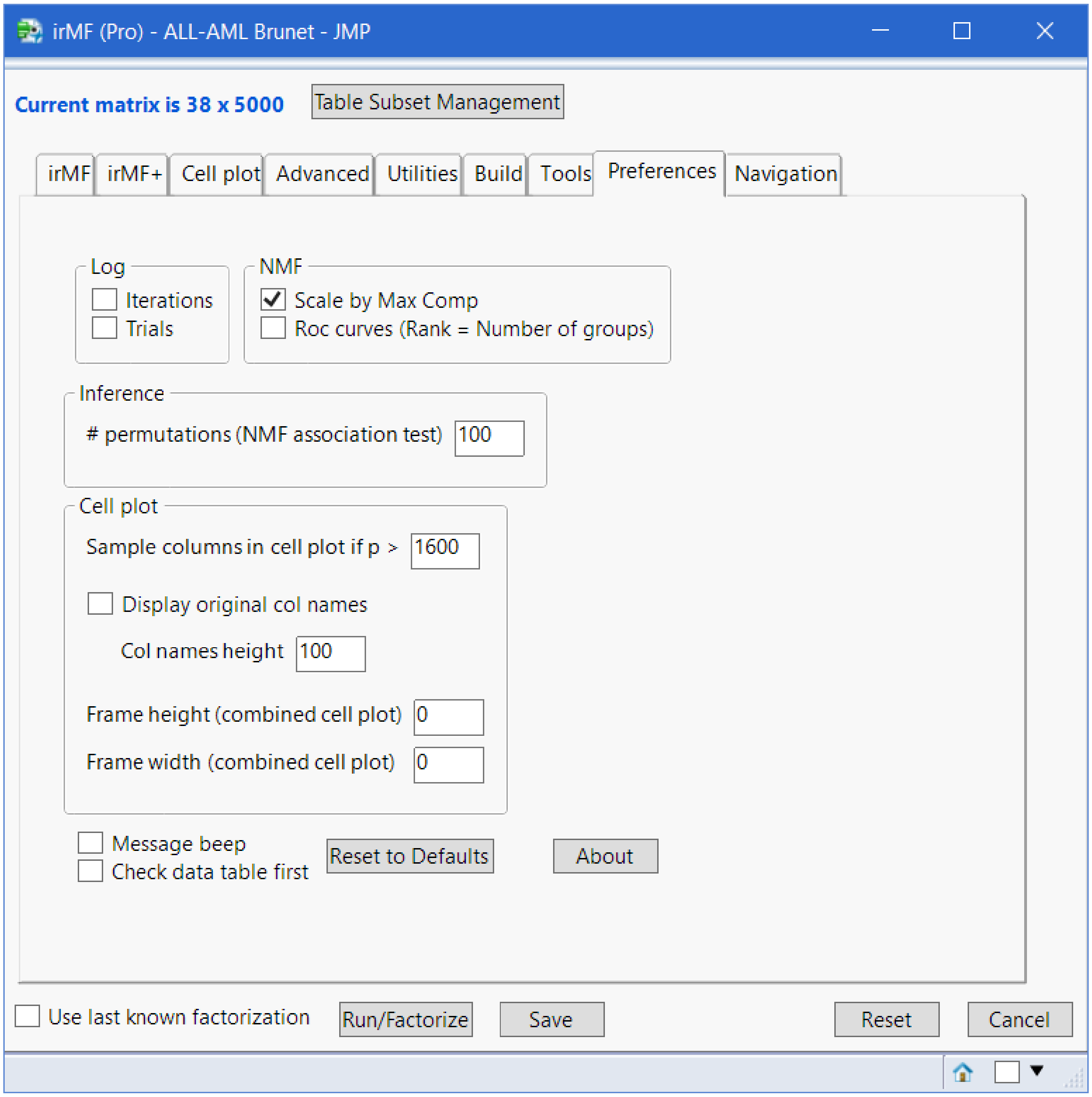

Note: Preference parameters are not stored in data table file as they are not specific to the dataset under study. When starting JMP (or after resetting irMF workspace), default parameters – as stored in file irMFini.jsl – are applied. This file can be retrieved by Windows search function and is editable but must be handled with care.

Iterations and trials residuals (Mean Square Error) can be logged in JMP log window.

NMF:

- **`Scale by Max Comp`**: By default, each left factoring vector is scaled by its max component. Uncheck this box to get back to the more usual L2 or L1 scaling (depending on the algorithm chosen).

- **Roc curves**: In the special case where the number of components is set equal to the number of groups, Roc curves are built for all pairs of components if the option is checked in the `Preferences` tab. For each pair, the discriminant function is the ratio of the second over the first left component. Recall that the Roc curve represents false versus true positive rates as a function of a cutoff in the discriminant function. In order to calculate these rates, each cluster must be associated with an experimental group. The cluster sample which has the highest LHE element points to the associated experimental group.

Inference:

# permutations (NMF association test): Set the number of runs which are performed during the permutation test used to assess the association between found NMF clusters and actual groups.

Sample columns: When the number of columns is too large to be displayed on a single page (due to screen resolution), a random sample of columns is displayed. Note that the calculations are done on the full data set.

Display original col names: Check this option to display column names as they appear in the table. Otherwise column numbers will be displayed. Note that if there are many columns, names will be unreadable.

Col names height: It may be useful to increase the default height to allow for displaying long variable names.

Frame height and width: Useful to adjust frame sizes of all parts of the combined cell plot.

Message beep: Checking this option will cause irMF messages to beep.

Check data table first: When checked, this option can substantially slow down the opening of irMF dialog when the number of rows in the data table is very large (e.g. > 1000). If unchecked, preliminary checks - like ensuring non-negativity before running NMF - are disabled.

Reset to default: irMF default parameters are defined in the jsl script file irMFini.jsl. Use a text editor to edit this file if you want to modify the defaults (however back up the original file before).

About: Clicking on this button opens the *About* window:

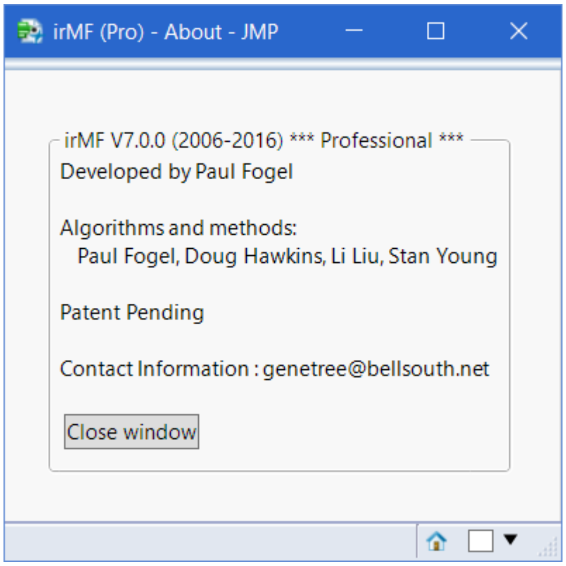

5.8 NAVIGATION

This tab allows for navigating across the different parts of irMF, namely: table, report and journal.

6 OUTPUTS

Scree plots, cell plots and Roc curves are all stored in a JMP report. By clicking on the red triangle on the top of the report, it is possible to either relaunch the analysis or to send first the report to the journal and relaunch the analysis)

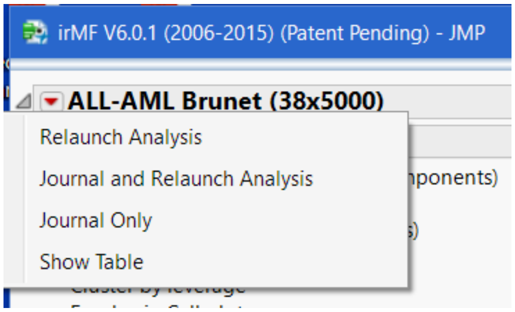

A number of tables are created:

- Scaling factors (Table name: `(irMF)Scaling factors`). These factors are all equal to 1 if NMF is applied since NMF factors are scaled in a particular way (see below).
- Left hand factoring vectors (Table name: `(irMF) Left factoring vectors`).
- Right hand factoring vectors (Table name: `(irMF) Right factoring vectors`). An additional column (baseline) is added if the NMF transform Min(col)=0 or Robust Min(col)=0 has been activated. This column corresponds to the min or robust min vector of original variables.

- Percent of outliers by row or column (SVD robust algorithm only, Table name: `(irMF) Marked outliers by column/row`).
- Additional tables used by cell plots.

Note: When working on a subset, the name of the subset is appended to all irMF table names. All irMF files are saved in the directory that contains the analyzed dataset, under a sub-directory after the name of dataset.

7 NOTES

Options are saved in a table script named `SetirMFoptions`. Save your dataset before closing it, to allow irMF retrieve automatically last used options whenever the dataset will be open again.

Modifying the `irMFini.jsl`: This script contains default parameters that will automatically appear in irMF dialogs the first time it is called with a given data table. These parameters can be changed by the user.

> Note: irMF preference parameters apply to all tables and are not saved in table script. Within a session, preference parameters can be changed though the preference dialog. To make these changes permanent, edit the `irMFini.jsl` file. Preference parameters appear on the top. You can change the values taken by any of the parameters, but the "`if (isEmpty(`…" conditional statements must not be changed.

8 A SMALL TUTORIAL

Open the jmp file `ALL-AML Brunet`; This dataset has 38 rows (samples) and 5000 numerical columns (genes). The first two columns contain samples id's and respected disease group – either ALL_B, ALL_T or AML (see Brunet, 2004, for a detailed description of the dataset).

In the main tab, choose the `Scree Plot` emphasis, enter 20 in the rank field, and run irMF:

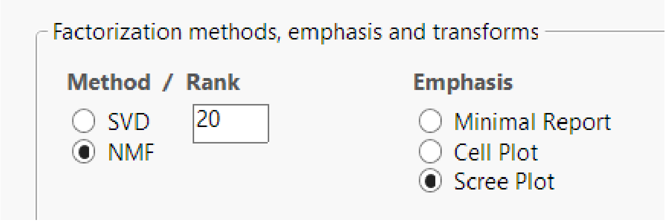

The irMF report contains several screeplots:

Warning! In 20-components model, cluster 17 has less than 3 members.

Warning! In 20-components model, cluster 20 has less than 3 members.

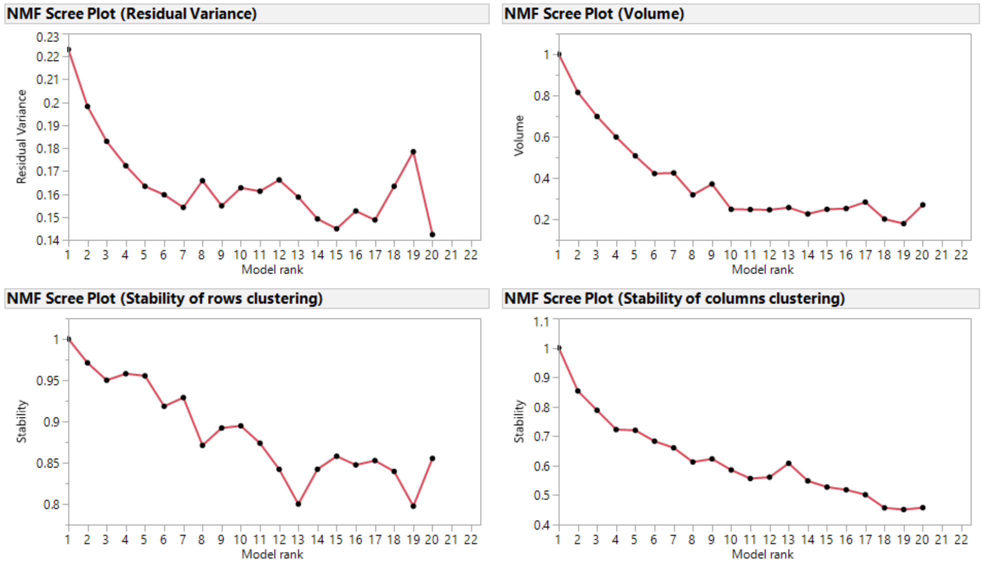

Let us focus on the two screeplots on the left:

- Residual variance becomes erratic when rank exceeds 7
- Stability of rows clustering indicates that rank 4 or 5 ensures a good stability

The stability measure is consistent with Brunet’s cophenetic correlation measure, indicating that 4 components improves the stability of the clustering. Since the number of disease types is only 3, this may suggest the existence of a disease subtype.

Note: the warnings indicate that clusters are nearly empty when the rank becomes too large.

Run NMF, this time with cell plot emphasis. Enter 4 in the rank field:

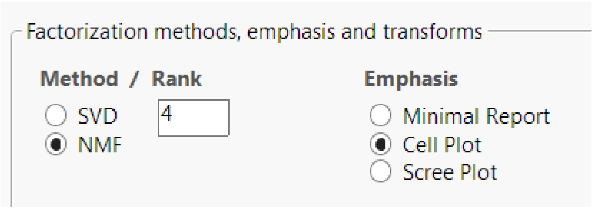

The irMF report contains several cell plot sections, however only the *Combined cell plot* is visible:

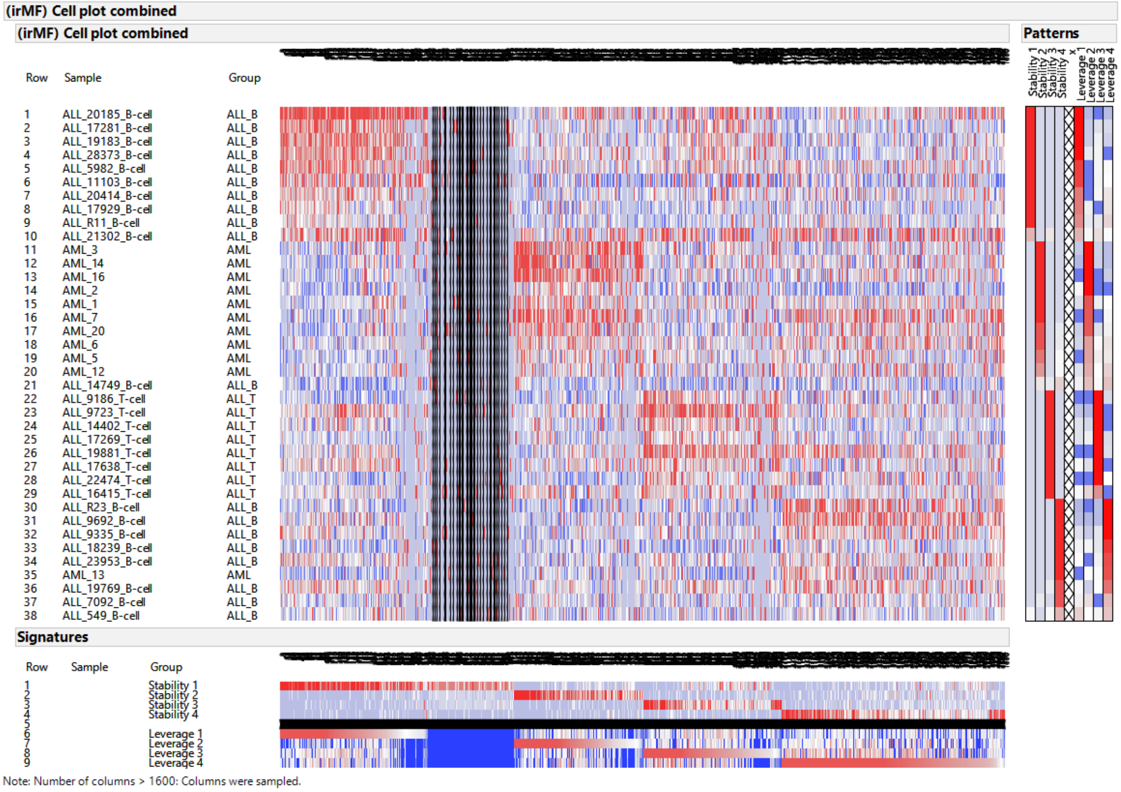

An association table between clusters and existing disease types appears under the cell plot:

Group size by cluster & significance of main group

[using hypergeometric distribution]

Cluster (Size Entropy)	ALL_B	ALL_T	AML
C 1 (10; 6.48)	10 [0.0002*]	0	0
C 2 (11; 6.42)	1	0	10 [<0.0001*]
C 3 (8; 6.55)	0	8 [<0.0001*]	0
C 4 (9; 6.48)	8 [0.0094*]	0	1

Weighted score: 34.47
Global significance using 100 permutations: 0.01

We note that clusters 1 and 4 are strongly associated with ALL_B disease type. In order to make these clusters contiguous, we change the components ordering entry in main irMF tab:

NMF components ordering

- ○ Use original order
- ○ Use scale
- ◉ Custom order

{1,4,3,2} (e.g. 1 3 2)

(note that `Use last know factorization` at the bottom of the dialog was checked automatically)

We obtain the reordered cell plot and association table:

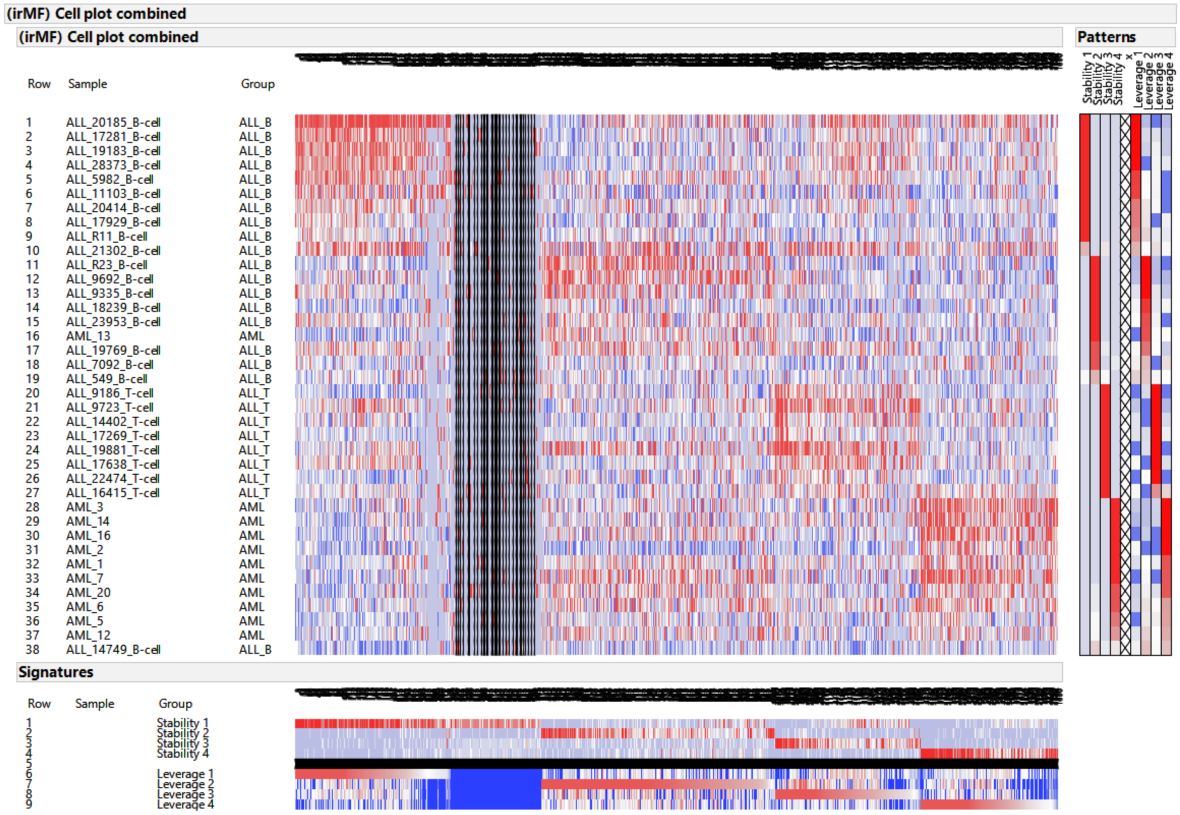

Note: Number of columns > 1600: Columns were sampled.

Group size by cluster & significance of main group

[using hypergeometric distribution]

Cluster (Size Entropy)	ALL_B	ALL_T	AML
C 1 (10; 6.48)	10 [0.0002*]	0	0
C 2 (9; 6.48)	8 [0.0094*]	0	1
C 3 (8; 6.55)	0	8 [<0.0001*]	0
C 4 (11; 6.42)	1	0	10 [<0.0001*]

Weighted score: 34.47
Global significance using 100 permutations: 0.01

The black columns in the cell plot indicates that some genes have zero variability (all samples have baseline values set at 20).

The NMF/Lasso will help get rid automatically of such obviously uninformative genes and will also cancel genes which appear to be redundant with respect to the left factoring vectors – called also variability patterns.

Open the main dialog and click on button `Lasso quick-setting` below the panel NMF options. The sparseness level automatically changes to 0.5, i.e. the Lasso penalty will be adjusted in order to cancel approximately 50% of the genes; The option `Use Trial LHE` is activated and the number of fixed LHE is set to 4 (= factorization rank); The report emphasis is set to `Minimal` and the chosen algorithm becomes `Least Squares`. NMF Lasso will now update the right factoring vectors accordingly.

Note that the button label `Lasso quick-setting` was automatically changed to `Standard quick-setting`. This will be useful, as we will see very soon.

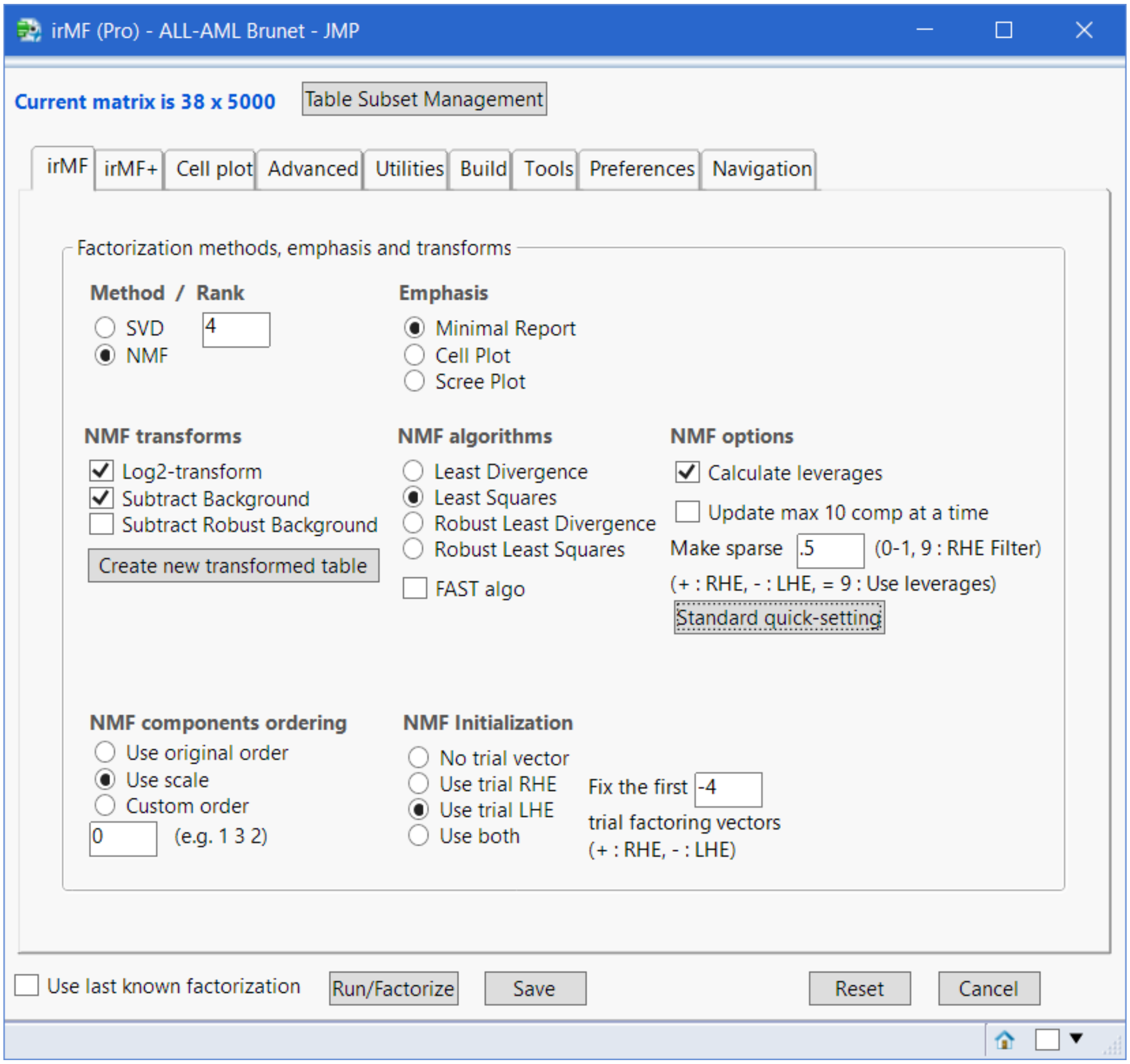

Run irMF; The status window displays the achieved level of sparseness. Once finished, the `Manage subset` dialog pops up automatically to save the subset with Lasso-selected genes.

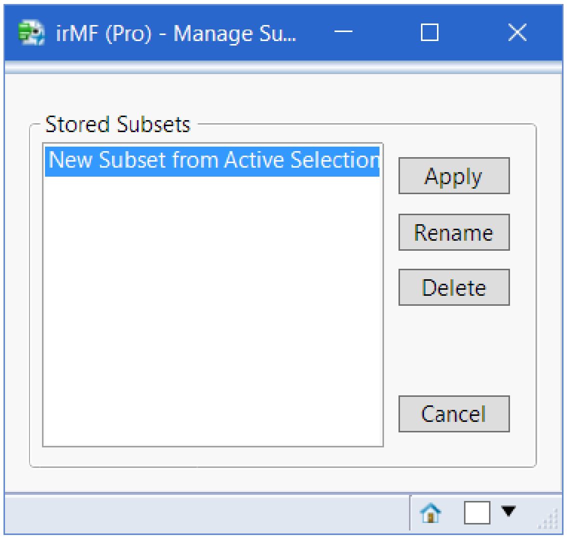

We enter the subset name: `1. SP50` to recall that the requested sparseness level was set to .50:

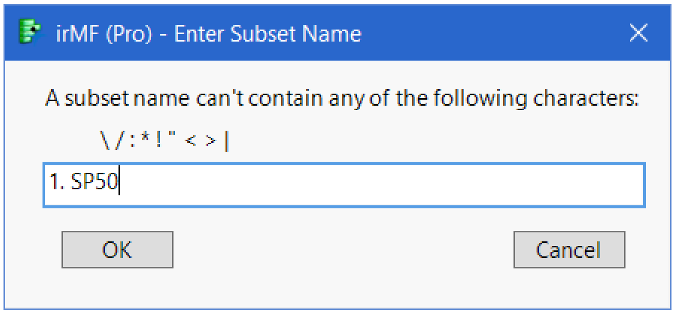

The main irMF dialog opens now; The new subset is active and has only 2476 genes (~50% of the initial 5000 genes).

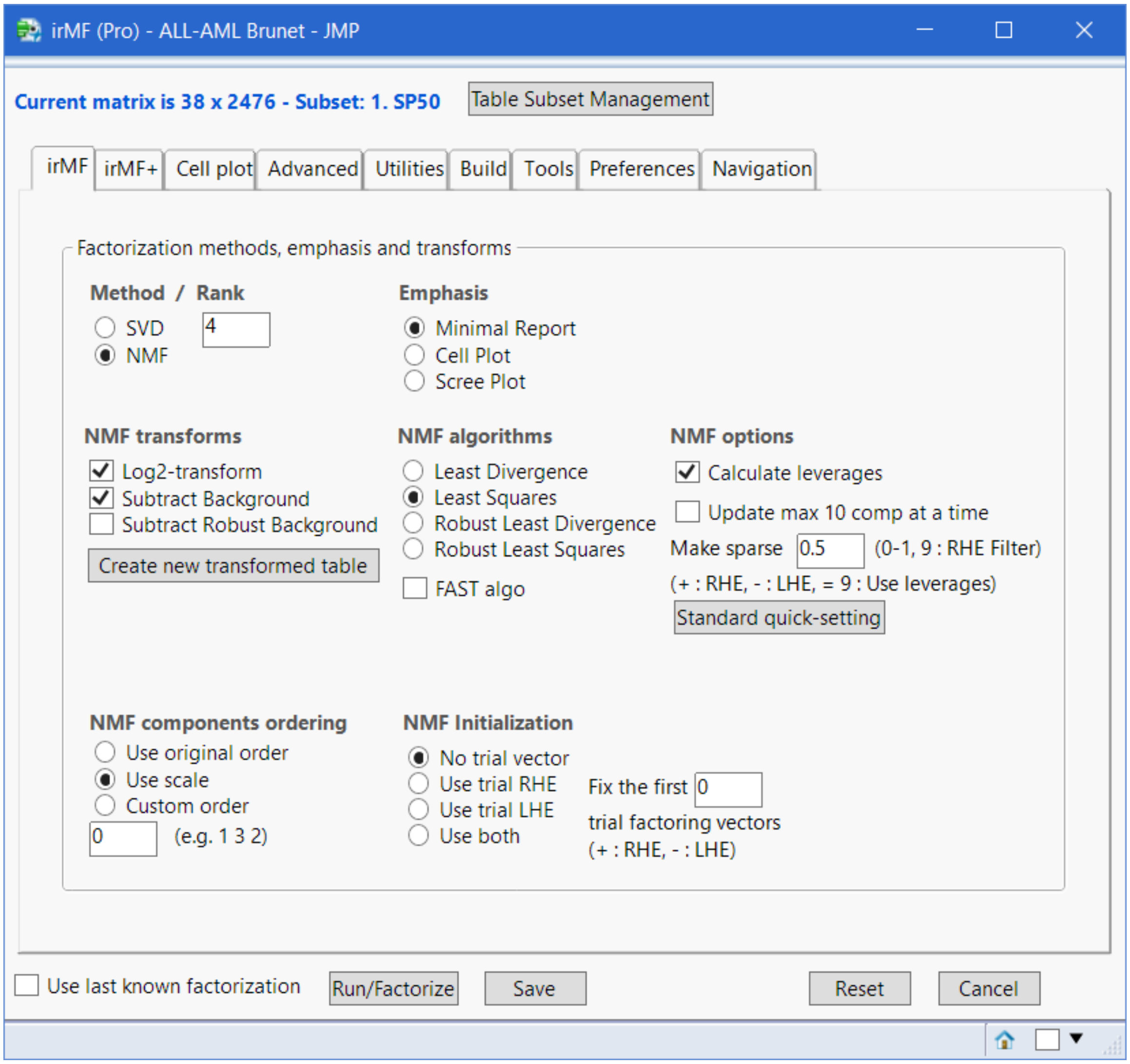

Click now on the button `Standard quick-setting` to restore the parameters to standard robust NMF and run irMF again. As with the analysis based on all genes, we note that clusters 1 and 4 are strongly associated with ALL_B disease type. In order to make these clusters contiguous, we change the components ordering entry in main irMF tab and obtain the following combined cell plot:

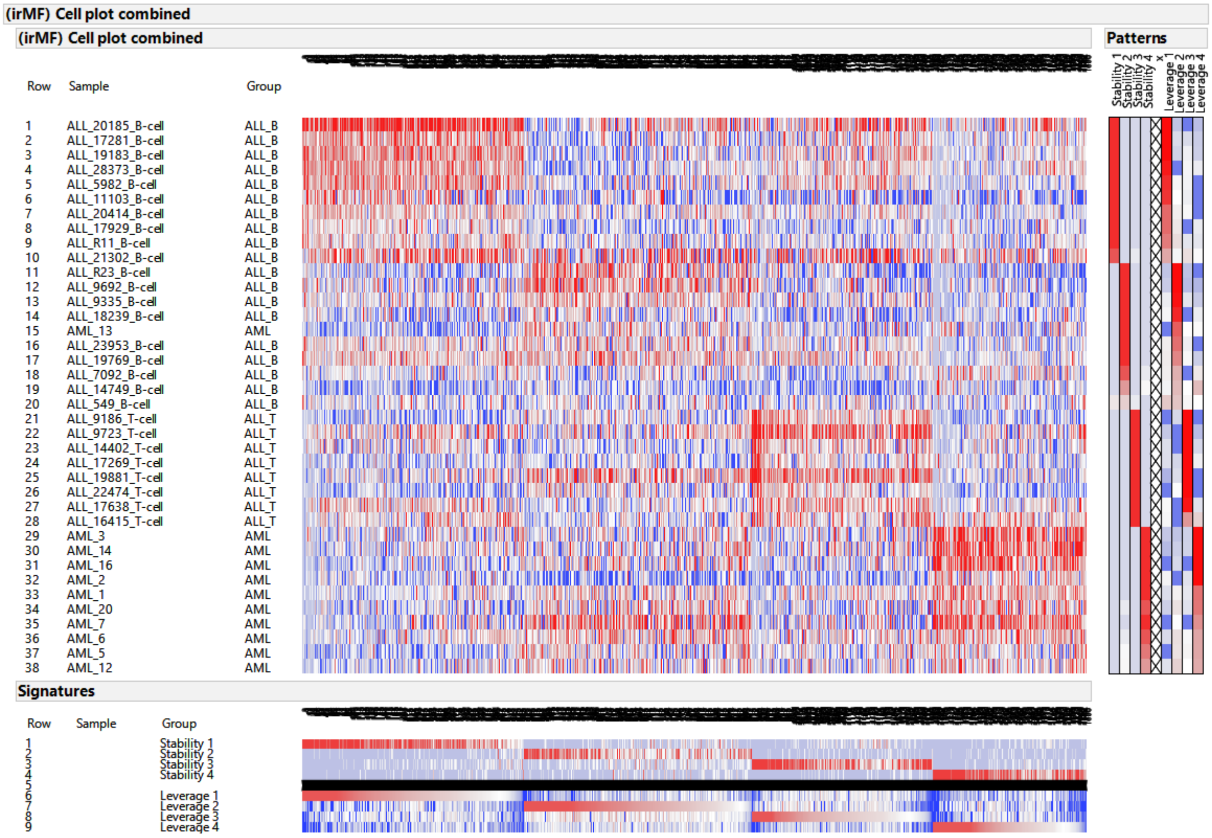

Note: Number of columns > 1600: Columns were sampled.

Note that the clustering is slightly improved as there is now only one error, caused by an AML sample – which is well known for being confused with other ALL_B disease samples.

Group size by cluster & significance of main group

[using hypergeometric distribution]

Cluster (Size Entropy)	ALL_B	ALL_T	AML
C1 (10; 8.28)	10 [0.0002*]	0	0
C2 (10; 8.26)	9 [0.0039*]	0	1
C3 (8; 8.34)	0	8 [<0.0001*]	0
C4 (10; 8.27)	0	0	10 [<0.0001*]

Weighted score: 36.13

Global significance using 100 permutations: 0.01

It is easy to check that the left factoring vectors associated with the Lasso subset are almost perfectly correlated with the original left factoring vectors. Choose the *Tools* tab and click on the button `Check Lasso patterns`. A dialog pops up, suggesting the name of the table where original left factoring vectors were stored:

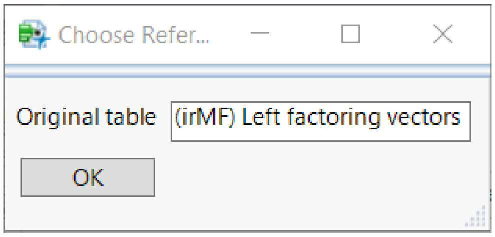

Open this table, which is stored under the sub-folder `(irMF) ALL-AML Brunet` and click *OK*. The scatterplot of Lasso versus Original left factoring vectors appears, confirming the strong correlation:

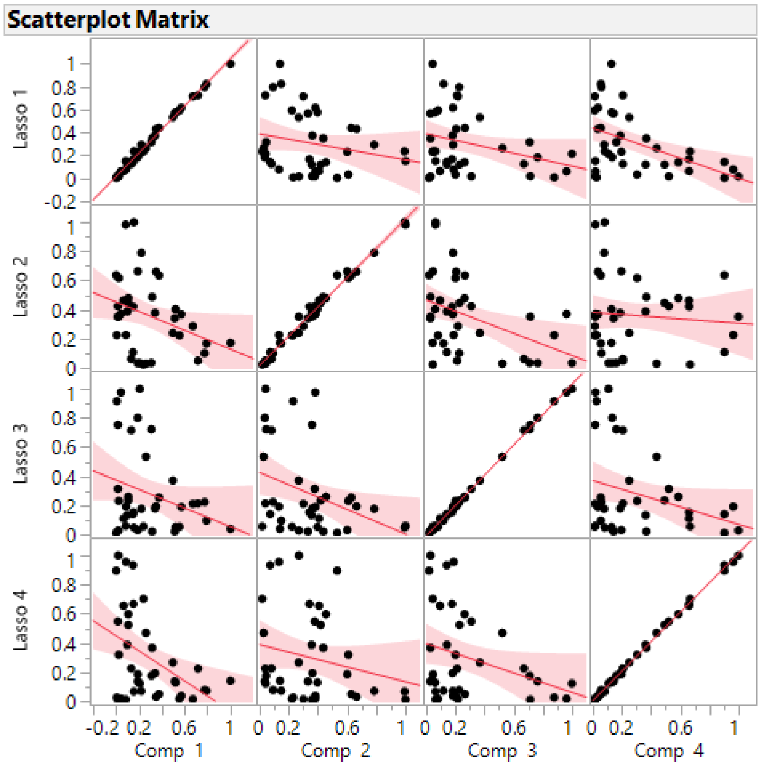

Of course, it is possible to cancel even more genes, as long as the resulting left factoring vectors remain almost unchanged, yielding a minimal subset of genes that carry the main signal found in the whole table.

On the contrary, it is possible to cancel a minimal number of genes – only the ones which do not contribute to the left factoring vectors. The procedure is a little more complex though:

First inverse the selection of Lasso columns to define a subset with the columns which are not selected by Lasso. Then run irMF and check that left factoring vectors are not well correlated with the original vectors as we did before with the Lasso selection:

Run irMF on the complementary subset of the 2476 genes selected by Lasso, and apply the above procedure to compare the new left factoring vectors with original ones:

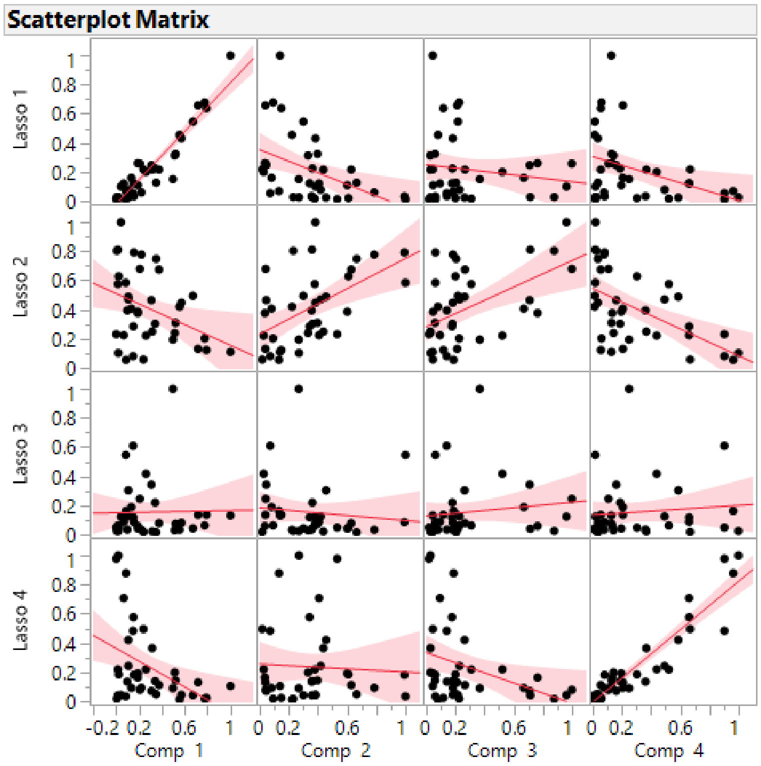

The scatterplot now shows that genes excluded by Lasso do not contain enough signal to retrieve the original left factoring vectors. Thus, the sparseness level can be set even higher without missing genes that contribute substantially to the original left factoring vectors.

Note that the `Check lasso-patterns` tool automatically fills the `Custom order` entry in the main table. This allows for further reordering the left factoring vectors, which are associated with the subset, in a way that best matches the order of the original vectors. Run again irMF with this Custom order setting to obtain a combined cell plot with samples ordering as close as possible to the original one.

Recall that the `Tools` tab contains functionalities that are still not part of the core irMF package, thus these somewhat cumbersome steps, which will be simplified in a future version

REFERENCES

Boutsisdis, C., and Gallopoulos, E. (2008) SVD Based Initialization: A Head Start for Nonnegative Matrix Factorization, *Journal of Pattern Recognition*, 41, 1350–1362.

Brunet, J.P., Tamayo, P., Golub, T.R., Mesirov, J.P. (2004). Metagenes and molecular pattern discovery using matrix factorization. *PNAS* 101, 4164–4169.

Devarajan, K. (2008). Nonnegative Matrix Factorization: An Analytical and Interpretive Tool in Computational Biology. *PLoS Computational Biology*, Vol. 4, Issue 7.

Lee, D.D., Seung H.S. (1999). Learning the parts of objects by non-negative matrix factorization. *Nature* 401, 788-791.

Liu, L., Hawkins, D.M., Ghosh, S., Young, S.S. (2003). Robust singular value decomposition analysis of microarray data. *PNAS* 100, 13167-13172.

Zhu,M. and Ghodsi,A. (2006). Automatic dimensionality selection from the scree plot

via the use of profile likelihood. *Comput. Stat. Data Anal.*, 51, 918–930.
